# Engineering defected 2D Pd/H-TiO_2_ nanosonosensitizers for hypoxia alleviation and enhanced sono-chemodynamic cancer nanotherapy

**DOI:** 10.1186/s12951-022-01398-6

**Published:** 2022-04-12

**Authors:** Xiaohui Qiao, Liyun Xue, Hui Huang, Xinyue Dai, Yu Chen, Hong Ding

**Affiliations:** 1 Department of Ultrasound, Huashan Hospital, Fudan University, Shanghai, 200040 People’s Republic of China; 2 Shanghai Engineering Research Center of Organ Repair, Materdicine Lab, School of Life Sciences, Shanghai University, Shanghai, 200444 People’s Republic of China

**Keywords:** Chemodynamic therapy, Nanozyme, Oxygen deficiency, Pd/H-TiO_2_ nanosheets, Sonodynamic therapy

## Abstract

**Background:**

Sonodynamic therapy (SDT) is a burgeoning modality for cancer therapy owing to its high tissue-penetrating capability, controllability and safety. Whereas, the undesirable reactive oxygen species (ROS) yield of sonosensitizers and tumor hypoxia are two vulnerable spots of SDT. Therefore, it is an advisable strategy to augment ROS level and simultaneously relieve hypoxia for SDT to arrive its full potential in cancer treatment.

**Results:**

In this work, the defected two-dimensional (2D) Pd/H-TiO_2_ nanosheets (NSs) with triple antineoplastic properties were dexterously elaborated and engineered using a facile one-pot Pd-catalyzed hydrogenation tactic by loading a tiny amount of Pd and then inletting hydrogen flow at atmospheric pressure and temperature. The 2D black Pd/H-TiO_2_ NSs with oxygen defects exerted eximious SDT effect based on the decreased bandgap that made it easier for the separation of electrons and holes when triggered by ultrasound as theoretically guided by density functional theory calculations. Additionally, Pd/H-TiO_2_ NSs could serve as Fenton-like agents because of the presence of oxygen defects, facilitating the conversion of hydrogen peroxide into hydroxyl radicals for exerting the chemodynamic therapy (CDT). Simultaneously, the introduced tiny Pd component possessed catalase-like activity responsible for oxygen production to ameliorate hypoxic condition and thus contributed to improving SDT and CDT efficacies. Both in vitro and in vivo results provided compelling evidences of high ROS yield and aggrandized sono-chemodynamic effect of Pd/H-TiO_2_ nanosonosensitizers with the detailed underlying mechanism investigation by RNA sequencing.

**Conclusion:**

This work delves the profound potential of Pd-catalyzed hydrogenated TiO_2_ on oncotherapy, and the effective antineoplastic performance and ignorable therapeutic toxicity make it a powerful competitor among a cornucopia of nanosonosensitizers.

**Graphical Abstract:**

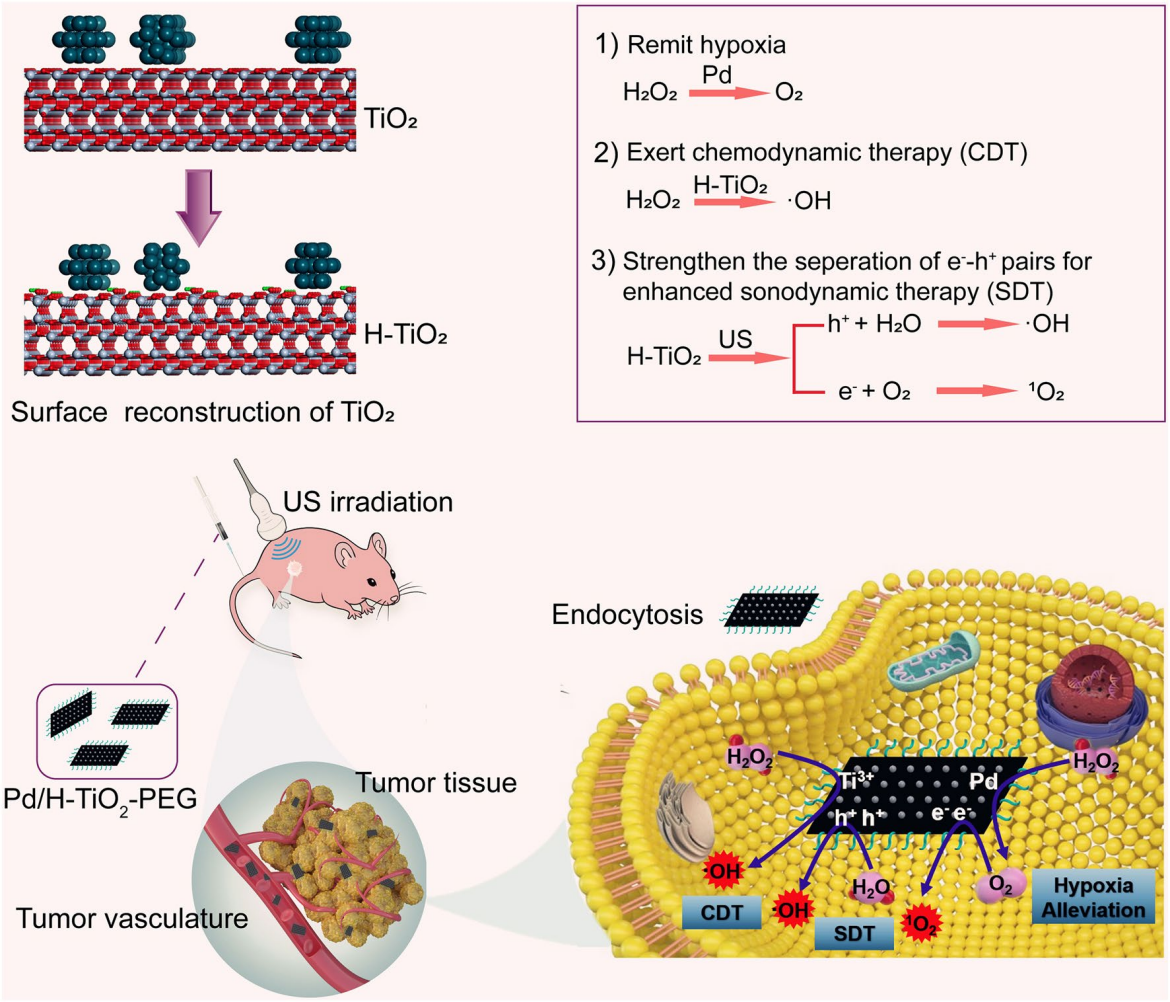

**Supplementary Information:**

The online version contains supplementary material available at 10.1186/s12951-022-01398-6.

## Introduction

Malignant tumor is one of the most serious worldwide causes of death and cancer therapy has always been a quandary that the medical profession has been wrestling with [1]. In view of the invasiveness, normal tissue damage and drug resistance that accompany surgery, chemotherapy, radiotherapy and targeted therapy [2–5], a therapeutic approach with remarkable efficacy and negligible side effects is desperately needed. The stimuli-responsive modalities initiated under the action of external and intratumoral incentives have been developing vigorously in cancer treatment owing to their site-confined lethality and controllability [6–8]. Wherein, sonodynamic therapy (SDT) is a burgeoning representative that takes ultrasound (US) as the external excitation source. When the tumor site is irradiated, sonosensitizers will be awakened accompanied by the generation of detrimental reactive oxygen species (ROS) to attack cancer cells [9–11]. Whereas, as a rising star, SDT has not absolutely arrived its potential because of the paucity of sonosensitizers with satisfactory response rate [12, 13]. Inorganic nanomaterials discard the shortcomings of inferior bioavailability, low stability and quick removal which are commonly seen in organic sonosensitizers, and feature the merits of tunable physicochemical property and preferable stability, thus have become promising candidates for sonosensitizers [14]. TiO_2_ itself can take the responsibility of generating ROS under sonication and can conjugate other functional constituents to accomplish multifunctionalization, so that stands out from numerous inorganic rivals [15–17]. However, due to the fast recombination of electrons (e^−^) and holes (h^+^), the ROS production of pure TiO_2_ nanoparticles is far from adequate for efficient SDT [18, 19]. Therefore, aggrandizing the quantity of e^−^-h^+^ pairs is one of the crucial strategies to improve SDT efficacy of TiO_2_-based sonosensitizers.

Chemodynamic therapy (CDT) is another emerging regimen of stimuli-responsive treatment modalities which is intelligently motivated by intrinsic incentives including acidity and hydrogen peroxide (H_2_O_2_) of tumor microenvironment to generate harmful hydroxyl radical (•OH) via Fenton or Fenton-like reaction [20–23]. Considerable researches have verified that in addition to Fe^2+^, various metal ions such as Mn^2+^, Cu^2+^ and Ti^3+^ can also act as Fenton-like agents and represent excellent catalytic properties [24]. In respect that both CDT and SDT are ROS-dependent tumor therapeutic paradigms, and delightedly, external sonication can promote •OH production by directly acting on some unstable molecules or through indirect thermogenesis hence boost CDT [24], the combination of CDT with SDT is undoubtedly an advisable cancer synergistic strategy. The booming development of nanotechnology can exactly build a bridge between CDT and SDT [25], a reasonable assembly of Ti^3+^-based Fenton-like agent and TiO_2_-based sonosensitizer can undertake the bilateral duty of CDT and SDT.

It should be recognized that hypoxia, the prevalent hallmark of the overwhelming majority of solid tumors induced by rapid proliferation and aberrant neovasculature, is a bottleneck for cancer treatment [26–28]. Definitely, the low oxygen tension not only contributes to tumor aggressiveness and metastasis but also results in compromised cytotoxic effect [29, 30]. On the one hand, as the donor of singlet oxygen (^1^O_2_), insufficient oxygen supply will inevitably impact the harvest of ROS and impair the potency of SDT [31]. On the other hand, oxygen depletion and vasculature damage during sono-chemodynamic therapy will further exacerbate oxygen deprivation, consequently, a vicious circle takes shape [32]. Therefore, it is sensible to replenish oxygen to relieve hypoxia and synchronously enhance SDT and CDT. Notably, the higher level of H_2_O_2_ in the tumor region can be decomposed into oxygen in the presence of the catalase accordingly can serve as feasible origination of oxygen to remit hypoxia [30, 33, 34]. And fortunately, diverse nanozymes that mimic natural enzyme capabilities have been extensively investigated and validated to be prospective alternatives to the scant autologous catalase in the tumor location [35, 36]. Overall, it is an alluring choice to employ the nanozyme in parallel with the sonosensitizer and Fenton/Fenton-like agent to purvey sufficient oxygen and contemporaneously enhance SDT and CDT.

**Scheme 1:**
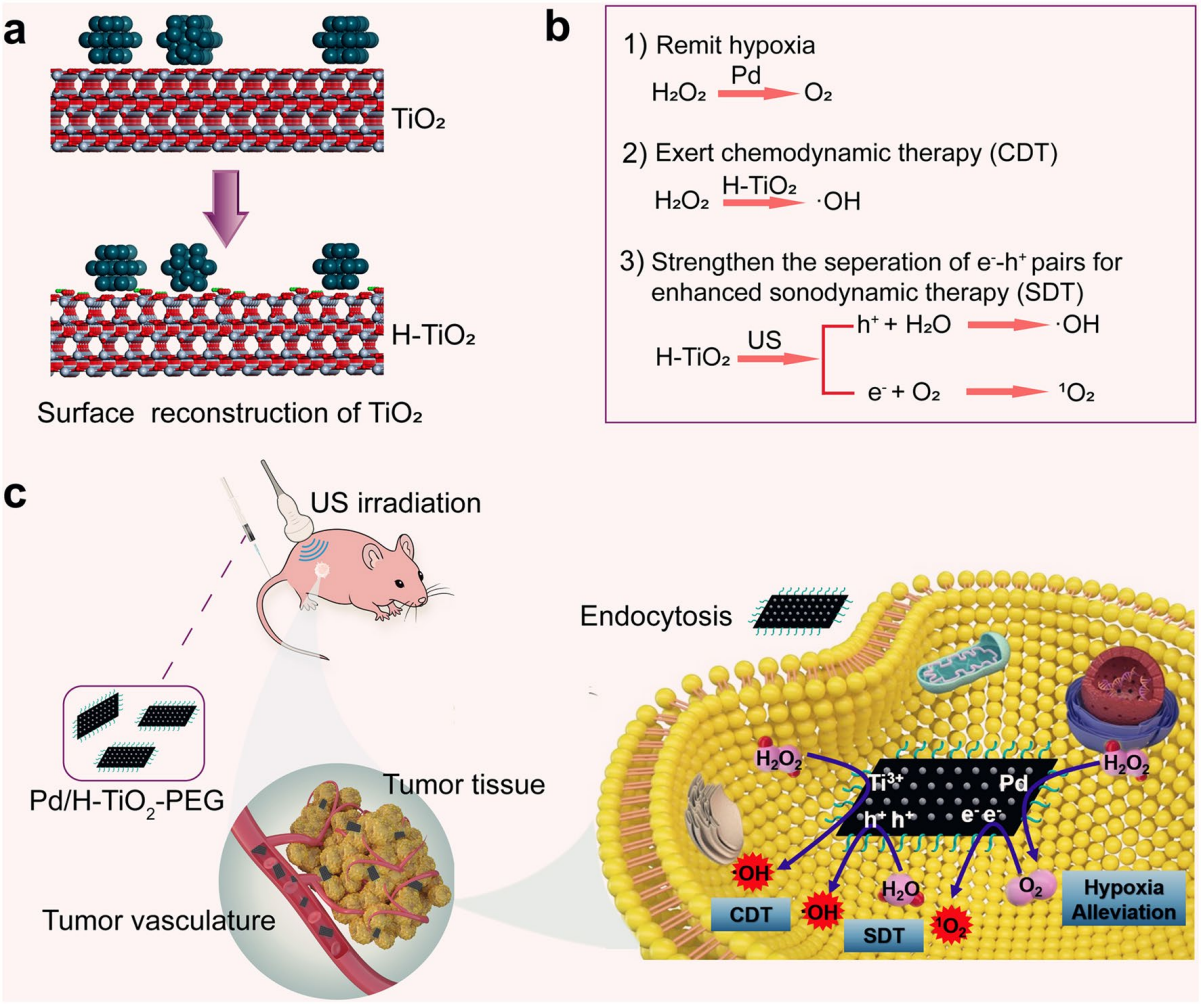
Schematic diagram of the antitumor mechanisms of Pd/H-TiO_2_-PEG for hypoxia alleviation and enhanced SDT and CDT. **a** Illustration of the surface reconstruction of TiO_2_ caused by H atom adsorption. **b** The multifunction of Pd/H-TiO_2_-PEG including yielding oxygen through Pd catalyzed decomposition of H_2_O_2_ for hypoxia amelioration, generating •OH through Ti^3+^ mediated Fenton-like reaction for CDT, and producing •OH as well as ^1^O_2_ through defected TiO_2_ sonosensitizer under US irradiation for enhanced SDT. **c** The in vivo hypoxia amelioration and synergetic enhancement of SDT and CDT for effective tumor suppression

Taking all above factors into consideration, we herein rationally designed and engineered the polyethylene glycol (PEG) modified dual stimuli-responsive Pd/H-TiO_2_-PEG nanosheets (NSs) as multifunctional nanosonosensitizers harboring one-arrow-three-hawks ability that could realize oxygen generation, enhanced SDT and CDT. For the nanocomposite, TiO_2_ matrixes were primitively synthesized, then thanks to the employment of Pd, the following H_2_ could naturally dissociate to atomic hydrogen and extend to TiO_2_ [37], this further led to the surface reconstruction and the formation of Ti^3+^ and oxygen defects (Scheme 1a). In this nanosystem, the defected TiO_2_ sonosensitizer, Ti^3+^ Fenton-like agent and Pd nanozyme coexist and have different ways to fulfill their duties. Admittedly, the existence of affluent defects can availably improve SDT efficacy of TiO_2_, since the defects can facilitate the creation of US incited e^−^-h^+^ pairs by declining the bandgap and hinder their recombination by trapping excited electrons [16, 38]. Apart from SDT, the nanoassembly is endowed with CDT function by provoking a Fenton-like reaction with the involvement of Ti^3+^. Interestingly, the US stimulus is also a favorable auxiliary method to speed up Fenton-like reaction to amplify CDT. Moreover, the attached Pd nanozyme makes a generous contribution to addressing the problem of hypoxia and providing ample oxygen source for ROS production by consuming endogenous H_2_O_2_ (Scheme 1b, c). The prominent cancer cell cytotoxicity and tumor suppression of enhanced SDT and CDT have been substantiated by in vitro and in vivo findings. Meanwhile, Pd/H-TiO_2_-PEG NSs have demonstrated inappreciable toxicity to the mice. This study displays a simple, mild and dexterous methodology for creating defects in TiO_2_ NSs, and the defected Pd/H-TiO_2_-PEG NSs are rendered synergistic multifunction for the forceful fight against cancer with reliable biosafety.

## Materials and methods

### Preparation of TiO_2_ NSs

10 mL of tetrabutyl titanate (TBT) and 1.2 mL of hydrofluoric acid (HF) were added into a 50 mL autoclave and adequately mixed under ultrasonication, followed by reaction at 180 °C for 24 h. When cooled, the product was centrifuged (8000 rpm, 10 min), washed with ethanol and deionized water and vacuum freeze-dried for subsequent procedures.

### Synthesis of Pd/H-TiO_2_-PEG

500 mg of TiO_2_ NSs were dispersed into 100 mL of ethylene glycol, after adding Na_2_PdCl_4_ solution (4 mg mL^−1^, 5 mL), the mixture was stirred for 6 h in the dark. Following additional 1 h of stirring with 1 g of polyvinylpyrrolidone (PVP), the solution was transferred into a flask to be heated to 150 °C while stirring intensely and then cooled down. Whereafter, the product (Pd/TiO_2_) was gathered after centrifugation, being washed with ethanol and deionized water, lyophilization and calcination at 300 °C for 2 h in air. Then, Pd/H-TiO_2_ was obtained by treating Pd/TiO_2_ with the H_2_/Ar (5 vol% H_2_) gas for 2 h in a tube furnace at atmospheric pressure and temperature. 30 mg of Pd/H-TiO_2_ and 300 mg of NH_2_-PEG_2000_ were dissolved into water and stirred for 48 h. Then, Pd/H-TiO_2_-PEG was obtained after being cleansed and re-dispersed into water.

### Oxygen release

A dissolved oxygen meter JPBJ-609L (Leici, China) was introduced to monitor the oxygen production after adding H_2_O_2_ (1 mM) into Pd/H-TiO_2_ or TiO_2_ solution with the same Ti concentration (400 μg mL^−1^). The numerical results were recorded every 30 s for 10 min. The US device M9cv with the L12-4S probe (Mindray, China) was selected to visualize oxygen production from pure H_2_O_2_, pure Pd/H-TiO_2_ and the mixture of Pd/H-TiO_2_ and H_2_O_2_.

### ^1^O_2_ generation

3 mL of Pd/H-TiO_2_ and TiO_2_ (50 μg mL^−1^ of Ti) were separately mixed with 60 μL of 1,3-diphenylisobenzofuran (DPBF, 1 mg mL^−1^), followed by 5 repetitions of US irradiation (1.5 W cm^−2^, 1.0 MHz, 50% duty cycle, 1 min) and ultraviolet–visible-near infrared (UV–vis-NIR) absorbance spectra scanning. The ^1^O_2_ yield was reflected by the absorbance decrease of DPBF at around 420 nm.

Moreover, the electron spin resonance (ESR) was implemented to ulteriorly favor the ^1^O_2_ generation by using 2,2,6,6-tetramethylpiperidine (TEMP) as the trapping agent. The groups included (1) Pd/H-TiO_2_, (2) H_2_O_2_, (3) Pd/H-TiO_2_ + H_2_O_2_, (4) US, (5) H_2_O_2_ + US, (6) Pd/H-TiO_2_ + US and (7) Pd/H-TiO_2_ + H_2_O_2_ + US. The concentrations of Ti and H_2_O_2_ were 200 μg mL^−1^ and 100 μM, respectively. 5 μL of TEMP was added into 200 μL of the total solution. The US irradiation time was 5 min (1.5 W cm^−2^, 1.0 MHz, 50% duty cycle).

###  •OH generation

3 mL of Pd/H-TiO_2_ and TiO_2_ (130 μg mL^−1^ of Ti) solutions were separately mixed with 130 μL of methylene blue (MB, 0.1 mg mL^−1^) solution followed by US irradiation and UV–vis-NIR absorbance spectra scanning. The •OH yield was reflected by the absorbance decrease of MB at around 664 nm. Besides, the generation of •OH from the mixture of Pd/H-TiO_2_ and H_2_O_2_ (100 μM) with or without US activation at pH 5.5 was detected in the same way.

ESR test was performed as well with 5,5-dimethyl-1-pyrroline N-oxide (DMPO) as the •OH trapping agent at pH 5.5, and 10 μL of DMPO was added into 200 μL of the total solution.

### SDT and CDT mediated by Pd/H-TiO_2_-PEG

Firstly, C6 cells were seeded in a 96-well plate and cultured overnight. Afterwards, the cells suffered the following disposals, including (1) Control, (2) Pd/H-TiO_2_-PEG, (3) H_2_O_2_, (4) Pd/H-TiO_2_-PEG + H_2_O_2_, (5) US, (6) H_2_O_2_ + US, (7) Pd/H-TiO_2_-PEG + US and (8) Pd/H-TiO_2_-PEG + H_2_O_2_ + US. The H_2_O_2_ (100 μM) and US treatments (1.5 W cm^−2^, 1.0 MHz, 50% duty cycle, 3 min) were operated after cells’ coincubation with Pd/H-TiO_2_-PEG (100 μg mL^−1^) for 4 h. Then the cells were continuously cultivated for 20 h and subjected to cell counting cit-8 (CCK-8, Dojindo Molecular Technologies, Inc.) assay. The viability of C6 cells suffering H_2_O_2_, US, and H_2_O_2_ + US disposal after coincubation with different concentrations of Pd/H-TiO_2_-PEG was also examined.

For live and dead cells visualization, C6 cells were inoculated in 6-well plates at 1 × 10^6^ cells per well overnight. After undergoing the aforementioned managements, cells were stained with calcein acetoxymethyl ester (Calcein-AM) and propidium iodide (PI) (Dojindo Molecular Technologies, Inc.) for 15 min, washed with phosphate buffer saline (PBS) and observed under the inverted fluorescence microscopy.

For cell apoptosis detection, C6 cells were treated with the same manners as mentioned above. After trypsin enzymic digestion, centrifugation, and resuspension with 500 μL of binding buffer, cells were stained with Annexin V-fluorescein isothiocyanate (FITC) and PI (Dojindo Molecular Technologies, Inc.) for 20 min and subjected to flow cytometry analysis.

### Intracellular ROS generation

C6 cells were seeded in 6-well plates and cultured overnight. After coincubation with Pd/H-TiO_2_-PEG (100 μg mL^−1^), discarding the medium and rinsing the cells, 2′,7′-dichlorofluorescin diacetate (DCFH-DA, 1 × 10^−6^ M) and H_2_O_2_ (100 μM) were added to the wells for 30 min. Then cells were washed twice gently and exposed to US irradiation (1.5 W cm^−2^, 1.0 MHz, 50% duty cycle, 3 min). Immediately, the cells were observed under the inverted fluorescence microscopy. Other comparisons were the same as above.

### Transcriptome analysis

C6 cells were seeded in a 6-well plate, three wells of which were set as experimental groups and cells were treated with H_2_O_2_ + US after coincubation with Pd/H-TiO_2_-PEG. And the other three wells were the control groups without any disposal. After extracting total RNA with TRIzol (Takara, Kyoto, Japan), the RNA samples were used for RNA sequencing by Shanghai Personal Biotechnology Co., Ltd. The data analysis was operated using R4.1.1 package "ggplot2" and through the free online platforms including http://www.bioinformatics.com.cn, https://www.string-db.org, and https://www.genescloud.cn (Personalbio GenesCloud).

### Animals

Healthy female ICR mice (5–6 weeks) and female BALB/c nude mice (4–5 weeks) were purchased from Jiangsu GemPharmatech Co., Ltd. All the animal procedures were carried out under the approval of the Ethic Committee of Shanghai University (ECSHU-2021-029).

### In vivo biodistribution

C6 cells (1 × 10^6^) suspended in 100 μL of PBS were subcutaneously injected into the right back of two female BALB/c nude mice. When the tumor maximum diameter was up to 10 mm the tumor block transplanting method was applied to establish the subcutaneous glioma model in 12 female BALB/c nude mice. Then 100 μL of Pd/H-TiO_2_-PEG (10 mg kg^−1^) was intravenously injected into the mice by the moment the tumor volume reached ~ 150 mm^3^, and the tumors as well as major organs were harvested at 4 h, 8 h, and 24 h (n = 4) post injection for weighting, digestion and Ti concentration test.

### In vivo anti-tumor therapy

Subcutaneous C6 glioma models were constructed in female BALB/c nude mice using the tumor block transplanting method. When the tumor volume was nearly 70 mm^3^, the mice were assigned into five groups (n = 5 in each group), including (1) Control (PBS, 100 μL, *i.v*. injection), (2) US (1.5 W cm^−2^, 1.0 MHz, 50% duty cycle, 3 min), (3) Pd/H-TiO_2_-PEG (10 mg kg^−1^, 100 μL, *i.v*. injection), (4) TiO_2_ + US (10 mg kg^−1^, 100 μL, *i.v*. injection, 1.5 W cm^−2^, 1.0 MHz, 50% duty cycle, 3 min) and (5) Pd/H-TiO_2_-PEG + US (10 mg kg^−1^, 100 μL, *i.v*. injection, 1.50 W cm^−2^, 1.0 MHz, 50% duty cycle, 3 min). US irradiation was performed at 24 h after injection. The body weight and tumor diameters were measured every other day until the 14th day. The mice were euthanized and tumors were resected for H&E, TUNEL, Ki-67, CD31 and HIF-1α analyses. The major organs were dissociated for H&E staining. The tumor volume was obtained by Eq. 1 and the tumor inhibition rate was calculated by Eq. 2.

1 $${\text{Tumor}}\,{\text{volume}}\, = \,{\text{length}}\, \times \,{\text{width}}^{2} /2$$

2 $${\text{Tumor}}\,{\text{inhibitation}}\,{\text{rate}}\, = \,\left( {{\text{1 - V}}_{{{\text{experiment}}}} {\text{/V}}_{{{\text{control}}}} } \right) \times 100\%$$

### Contrast enhanced ultrasound (CEUS) imaging

To visibly unfold the tumor vascularity, CEUS imaging was performed on the tumor-bearing mouse after being anesthetized with 4% chloral hydrate (10 μL g^−1^, i.p.) and injected 100 μL of contrast agent sulfur hexafluoride microbubble (SonoVue, Bracco Co., Italy) through the tail vein. For intratumoral oxygen generation, the tumor-bearing mouse was intratumorally injected with 50 μL of Pd/H-TiO_2_-PEG (1 mg mL^−1^), CEUS imaging was performed pre-injection, 20 and 40 min after injection.

### Statistical analysis

The data were presented as mean ± standard deviation. The one-way analysis of variance was applied for comparisons of multiple sets of data (*p < 0.05, **p < 0.01, ***p < 0.001).

## Results and discussion

### Design, preparation and characterization of Pd/H-TiO_2_ nanosonosensitizers

**Fig. 1:**
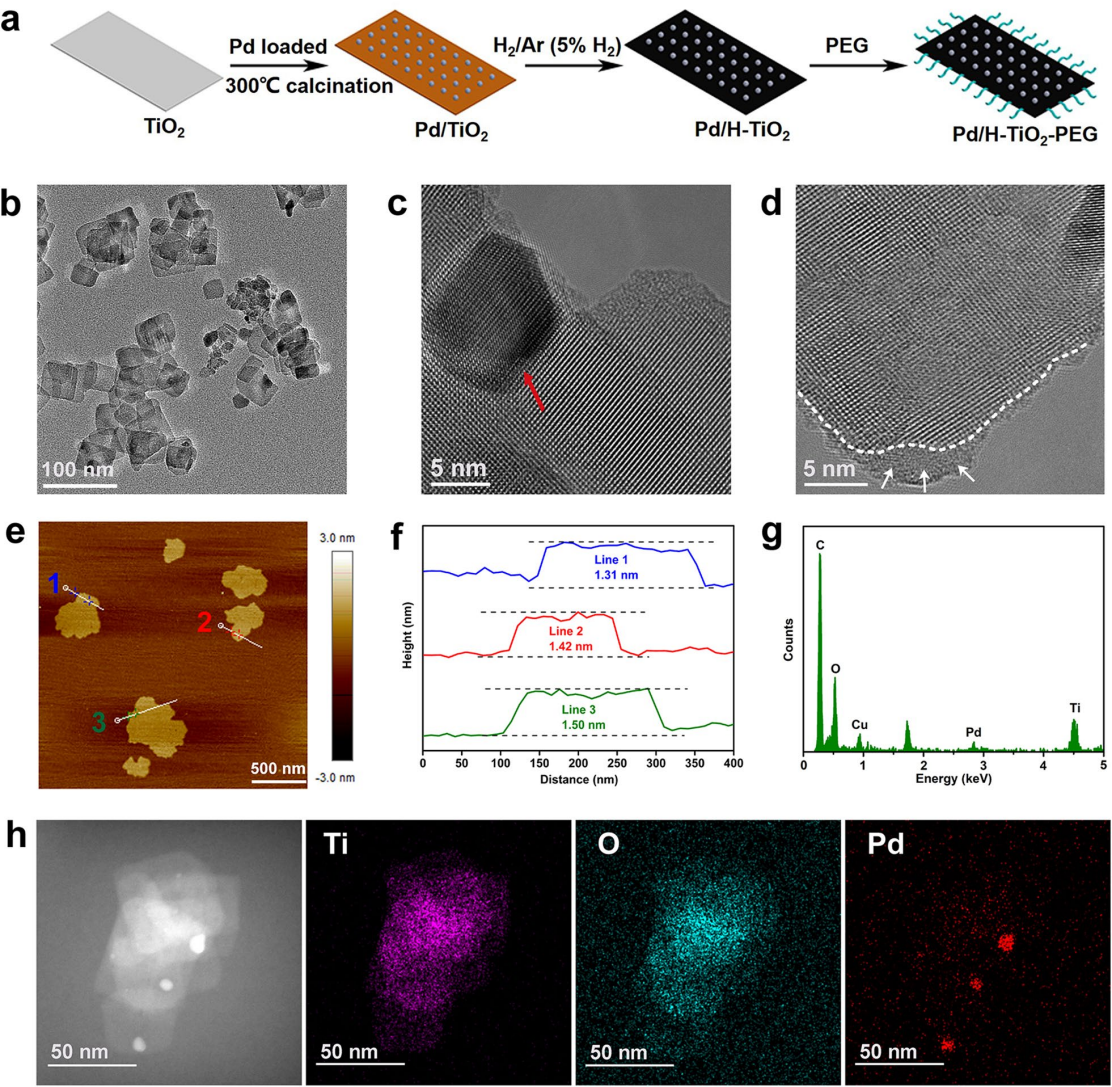
Synthetic scheme and structural characterization of Pd/H-TiO_2_. **a** Presentation of the preparation and surface PEGylation modification process of Pd/H-TiO_2_. **b** TEM and **c**, **d** HRTEM images of Pd/H-TiO_2_. **e** AFM image and **f** the corresponding thickness of Pd/H-TiO_2_ nanosheets. **g** EDS of Pd/H-TiO_2_. **h** Elemental analysis of Ti, O and Pd in Pd/H-TiO_2_

The Pd/H-TiO_2_ NSs were fabricated using a facile and mild hydrogenation maneuver according to the literature [37]. In detail, the regular white TiO_2_ NSs were initially synthesized via a simple hydrothermal route. Next, just a slight amount of Pd was incorporated on the surface of TiO_2_ to form Pd/TiO_2_. Afterwards, H_2_ flow was fed without involvement of harsh terms including particularly high pressure and temperature and excitingly the yellow Pd/TiO_2_ transformed into black Pd/H-TiO_2_ (Fig. 1a and Additional file 1: Figure S2). Eventually, NH_2_-PEG_2000_ was adopted for surface modification of Pd/H-TiO_2_ to improve the dispersity and biocompatibility.

It could be seen from the transmission electron microscope (TEM) image that Pd/H-TiO_2_ NSs were of uniform rectangular nanostructure with the average diameter of 61.9 nm (Fig. 1b and Additional file 1: Figure S3). Although only a small quantity of Pd was introduced in the nanosystem, it was distinctly espied on TiO_2_ substrate (Fig. 1c), indicating that Pd could be tightly anchored on TiO_2_ matrix. From TEM images, both Pd/H-TiO_2_ and TiO_2_ were rectangular in shape (Fig. 1b and Additional file 1: Figure S4a), but from high-resolution transmission electron microscope (HRTEM) images, the superficial structure of Pd/H-TiO_2_ markedly differed from TiO_2_. Compare with the well-crystallized TiO_2_ (Additional file 1: Figure S4b), Pd/H-TiO_2_ had a typical amorphous shell (Fig. 1d) which was ascribed to the existence of oxygen defects. This could be also verified by scanning electron microscope (SEM) images, from which we could see the sharp edge of TiO_2_ NSs (Additional file 1: Figure S5) while the margin of Pd/H-TiO_2_ NSs was blunter (Additional file 1: Figure S6). The thickness of Pd/H-TiO_2_ analyzed by atomic force microscope (AFM) was about 1.3–1.5 nm (Fig. 1e, f). The energy dispersive spectrometer (EDS) in conjunction with elemental mapping clearly showed the coexistence of titanium (Ti), oxygen (O) and palladium (Pd) elements in Pd/H-TiO_2_ (Fig. 1g, h), and no Pd element in pristine TiO_2_ (Additional file 1: Figure S4c).

**Fig. 2:**
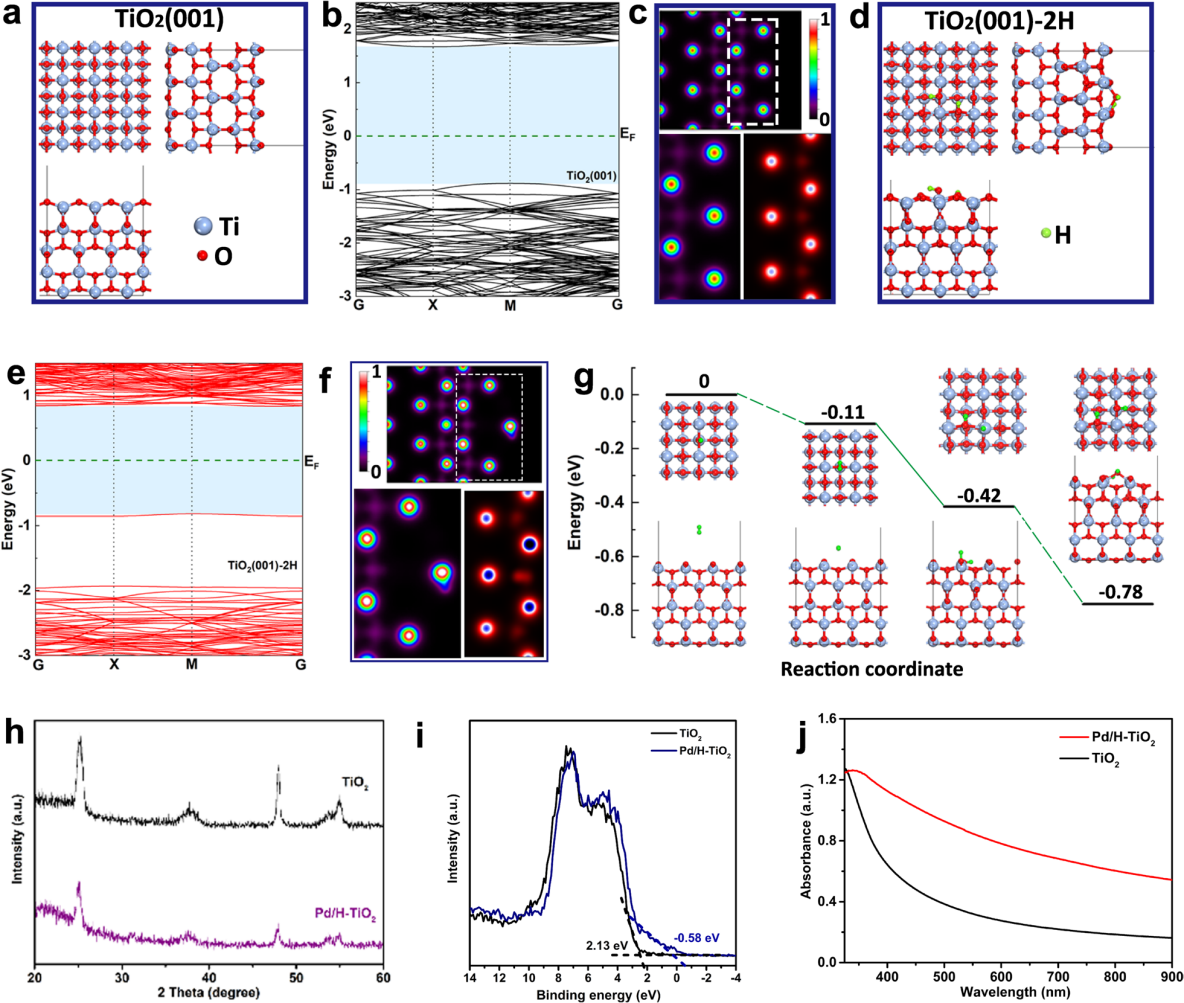
Electronic property based on DFT calculations, XRD, XPS-valence band and UV-vis-NIR analyses. Schematics, electronic band structures as well as 2D charge density maps of the **a-c** anatase TiO_2_(001) and **d-f** anatase TiO_2_(001)-2H. Blue, red and green balls indicated the Ti, O and H atoms, respectively. G, X and M in the band structures corresponded to the (0, 0, 0), (0.5, 0, 0) and (0.5, 0.5, 0) *k*-points in the Brillouin zone. The horizontal dashed line shows the Fermi level. **g** Energy diagram of the H_2_ adsorption process on the TiO_2_(001) surface (relative to the initial state energy). **h** XRD patterns of TiO_2_ and Pd/H-TiO_2_. **i** XPS- valence band spectra of TiO_2_ and Pd/H-TiO_2_. **j** The UV-vis-NIR spectra of TiO_2_ and Pd/H-TiO_2_

To shed light on the internal mechanism of the effect of H_2_ treatment on anatase TiO_2_, we performed density functional theory calculations with the Hubbard correction (DFT + U) on the electronic structures of the anatase TiO_2_(001) and TiO_2_(001)-2H (Additional file 1: Figure S1). The top, front and side views of these two models were shown in Fig. 2a, d, from which we could conclude that the adsorption of H atoms on the surface led to the deformation and reconstruction of the top two layers. Moreover, we plotted their 2D charge density maps at the same x coordinate position (Fig. 2c, f) to compare their density distribution difference. Obviously, the electrons rearranged because of the reconstructed top two layers, especially accumulated on the O atoms around H atoms. This phenomenon resulted in a new electronic band appearing at the middle of the bandgap of the TiO_2_(001)-2H compared with the original TiO_2_(001), which narrowed the bandgap from 2.56 eV to 1.66 eV and the long wave absorption was enhanced correspondingly (Fig. 2b, e). Simultaneously, we also calculated the total energy of H_2_ adsorption process on the TiO_2_(001) surface to verify the possibility of hydrogen atom adsorption. As shown in Fig. 2g, for the initial structure, there was a H_2_ molecule far away from the TiO_2_(001) surface. The H_2_ molecules were then moved toward the surface with a slight energy drop of 0.11 eV. When the H_2_ molecule was dissociated into two H atoms, one H was connected to the O atom, resulting in the deformation of the surface structure near the O, while the other H was connected to the adjacent Ti, and the energy drop of this process was 0.31 eV. When the second H atom desorbed from the Ti atom and then connected with the other nearest neighbor O atom, it would bring a higher energy drop (about 0.36 eV) and lead to more significant atomic reconstruction. These three energy drops demonstrated that it was energetically favorable of the reconstruction of the top two layers resulted from the adsorption of H_2_ molecules on the TiO_2_(001) surface.

Accordingly, the change of the electronic band structure did induce the differences of characterizations between Pd/H-TiO_2_ and TiO_2_. As suggested by X-ray diffraction (XRD) analysis, the diffraction peaks of Pd/H-TiO_2_ were in line with those of TiO_2_ but the peak heights dropped markedly (Fig. 2h), implying that the essential crystalline structure of Pd/H-TiO_2_ remained unchanged while the crystallinity decreased thanks to the formation of the amorphous layer. As indicated by X-ray photoelectron spectroscopy (XPS) analysis, the peak positions of Ti 2p (Additional file 1: Figure S7a, b), O 1 s (Additional file 1: Figure S7c, d) and C 1 s (Additional file 1: Figure S7e, f) in TiO_2_ and Pd/H-TiO_2_ were basically coincident, illustrating their similar binding energies with the environment. Additional file 1: Figure S8 disclosed the emergence of high valence state of Pd, which may be pertinent to the dissociation of H_2_. Specially, the valence band spectra uncovered the blue-shift of the valence band maximum of Pd/H-TiO_2_ (Fig. 2i), justifying the narrowed bandgap of Pd/H-TiO_2_. Besides, the narrowed bandgap of Pd/H-TiO_2_ was proved by the UV–vis-NIR absorbance spectra as well, from which the higher optical absorption of Pd/H-TiO_2_ over a wide wavelength range was readily observed (Fig. 2j). These results together revealed the process of TiO_2_ hydrogenation, that is, the process of white TiO_2_ turning into black Pd/H-TiO_2_. Specifically, the employed Pd induced the dissociation of H_2_ molecules on its surface, and then the dissociated hydrogen species spread to TiO_2_, reducing Ti^4+^ to Ti^3+^ accompanied by the generation of oxygen deficiencies. This process further resulted in the formation of a middle electronic band which finally narrowed the bandgap of TiO_2_ and augmented the visible-light absorption.

### In vitro SDT, CDT and nanozyme efficiency of Pd/H-TiO_2_ nanosonosensitizers

**Fig. 3:**
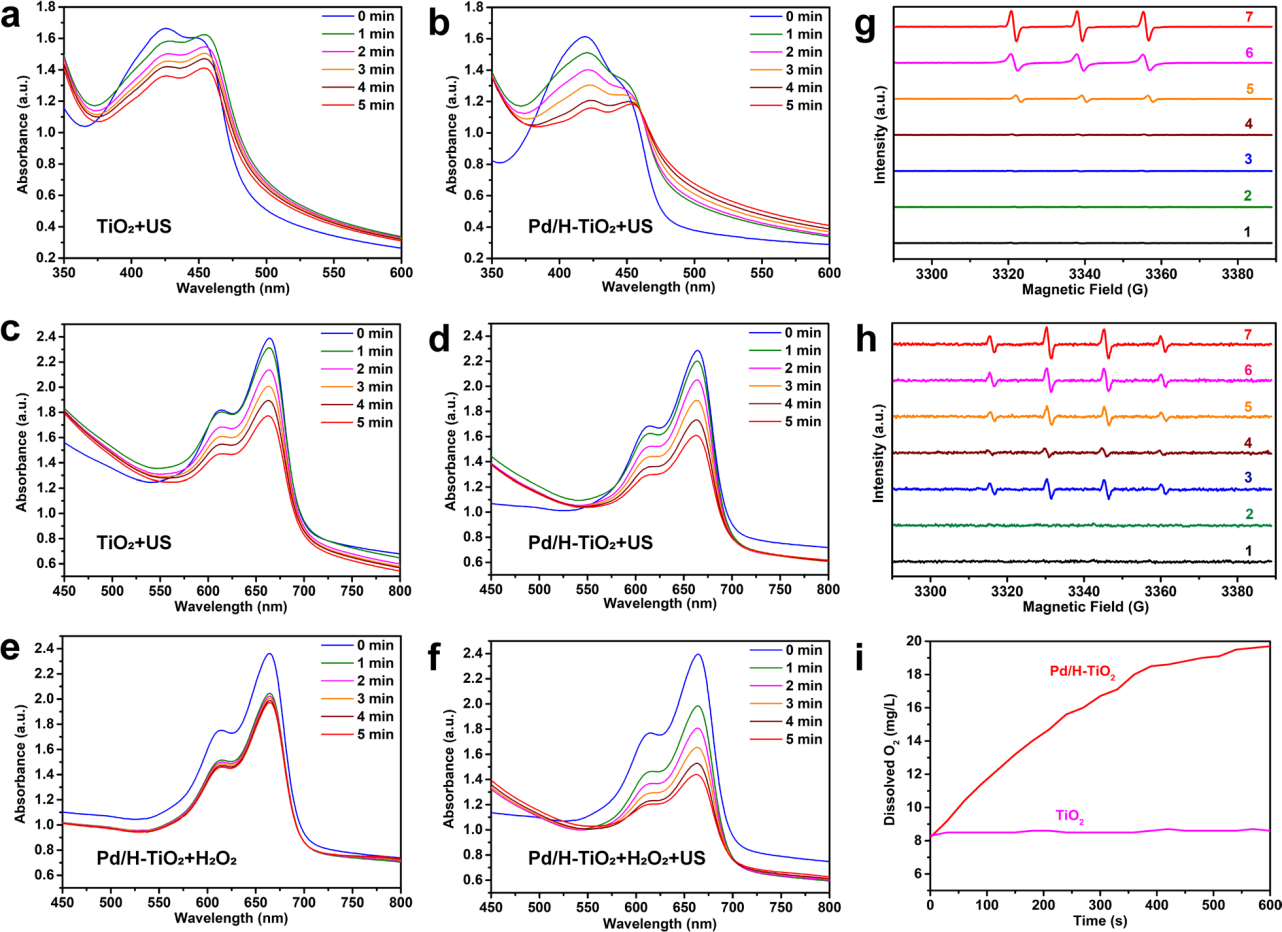
In vitro ROS and oxygen generation capabilities of Pd/H-TiO_2_. **a** TiO_2_ and **b** Pd/H-TiO_2_ mediated SDT performance demonstrated by the degradation of DPBF. **c** TiO_2_ and **d** Pd/H-TiO_2_ mediated SDT performance demonstrated by the degradation of MB. **e** CDT performance of Pd/H-TiO_2_ demonstrated by the degradation of MB. **f** Enhanced SDT and CDT performance of Pd/H-TiO_2_ demonstrated by the degradation of MB. Detection of **g** ^1^O_2_ and **h** •OH generated from different solutions through ESR spectra. The groups were: (1) Pd/H-TiO_2_, (2) H_2_O_2_, (3) Pd/H-TiO_2_ + H_2_O_2_, (4) US, (5) H_2_O_2_ + US, (6) Pd/H-TiO_2_ + US and (7) Pd/H-TiO_2_ + H_2_O_2_ + US. **i** Dissolved oxygen generation of TiO_2_ and Pd/H-TiO_2_ in H_2_O_2_ solution

Inspired by the existence of oxygen deficiencies and narrowed bandgap of Pd/H-TiO_2_ nanosonosensitizers, the SDT and CDT performances were then explored by evaluating the generation of ROS including ^1^O_2_ and •OH. For SDT performance, the ^1^O_2_ formed through the reaction of e^−^ and oxygen molecules under US stimulation was initially detected using DPBF as the probe, based on the fact that the characteristic absorption peak of DPBF at around 420 nm in the UV–vis-NIR spectrum would decrease in the presence of ^1^O_2_. As expected, after irradiating the mixture of DPBF and Pd/H-TiO_2_, the absorbance intensity of DPBF apparently declined along with the extension of exposure time, and the descend range was wider in comparison with the mixture of DPBF and TiO_2_ at the same conditions (Fig. 3a, b). It could be seen from the results that Pd/H-TiO_2_ manifested enhanced efficiency of ^1^O_2_ production which was attributed to the disordered layer with abundant oxygen defects. Apart from ^1^O_2_, •OH was another member of ROS family, forming through the reaction of h^+^ and the surrounding water molecules under US excitation. The MB was chosen as the probe of •OH, for its degradation in the solution containing •OH would lead to the fall of the absorption peak at around 664 nm. Likewise, the •OH content in Pd/H-TiO_2_ solution was pronouncedly higher than that in TiO_2_ solution (Fig. 3c, d). The overt high yield of ^1^O_2_ and •OH guaranteed the eximious SDT performance of Pd/H-TiO_2_. On account of the formation of oxygen defects, it was presumed that there would be the coexistence of Ti^3+^ which could participate in Fenton-like reaction making contributions to •OH generation. Unsurprisingly, the downtrend of the absorption peak of MB was assuredly espied when Pd/H-TiO_2_ was blended with H_2_O_2_ at pH 5.5 (Fig. 3e), proving its CDT ability. And peculiarly, the downward tendency was more pronounced when US was imposed at the same conditions (Fig. 3f), suggesting the synchronous augment of SDT and CDT. The bilateral enhancement of SDT and CDT was further supported by ESR with TEMP and DMPO as the trapping agent of ^1^O_2_ and •OH, respectively. It was found that the maximum signal intensity appeared in Pd/H-TiO_2_ + H_2_O_2_ + US group no matter for ^1^O_2_ or •OH (Fig. 3g, h). The outcomes were consistent with that of UV–vis-NIR spectra to jointly confirm the reinforced SDT and CDT of Pd/H-TiO_2_.

As a type of precious metal, Pd has been comprehensively applied in biological areas considering its enzyme-mimetic activities [39–41]. Here, its catalase-like ability which meant the ability of catalyzing H_2_O_2_ into oxygen was tested by using a dissolved oxygen meter to monitor the change of oxygen concentration after adding H_2_O_2_ into Pd/H-TiO_2_ and TiO_2_ solutions under stirring. As shown in Fig. 3i, the oxygen concentration in Pd/H-TiO_2_ solution was constantly rising with time while there was no noticeable uptrend of oxygen concentration in TiO_2_ solution. Furthermore, CEUS was utilized to intuitively display oxygen production from H_2_O_2_, Pd/H-TiO_2_ and Pd/H-TiO_2_ + H_2_O_2_ solutions, for bubbles’ excellent backscattering capability could remarkably intensify the echo signal [42–44]. We observed the most obviously enhanced signal in Pd/H-TiO_2_ + H_2_O_2_ group from Additional file 1: Figure S9, which vividly substantiated the catalase-like capability of Pd/H-TiO_2_.

### In vitro SDT and CDT effects against tumor cells

**Fig. 4:**
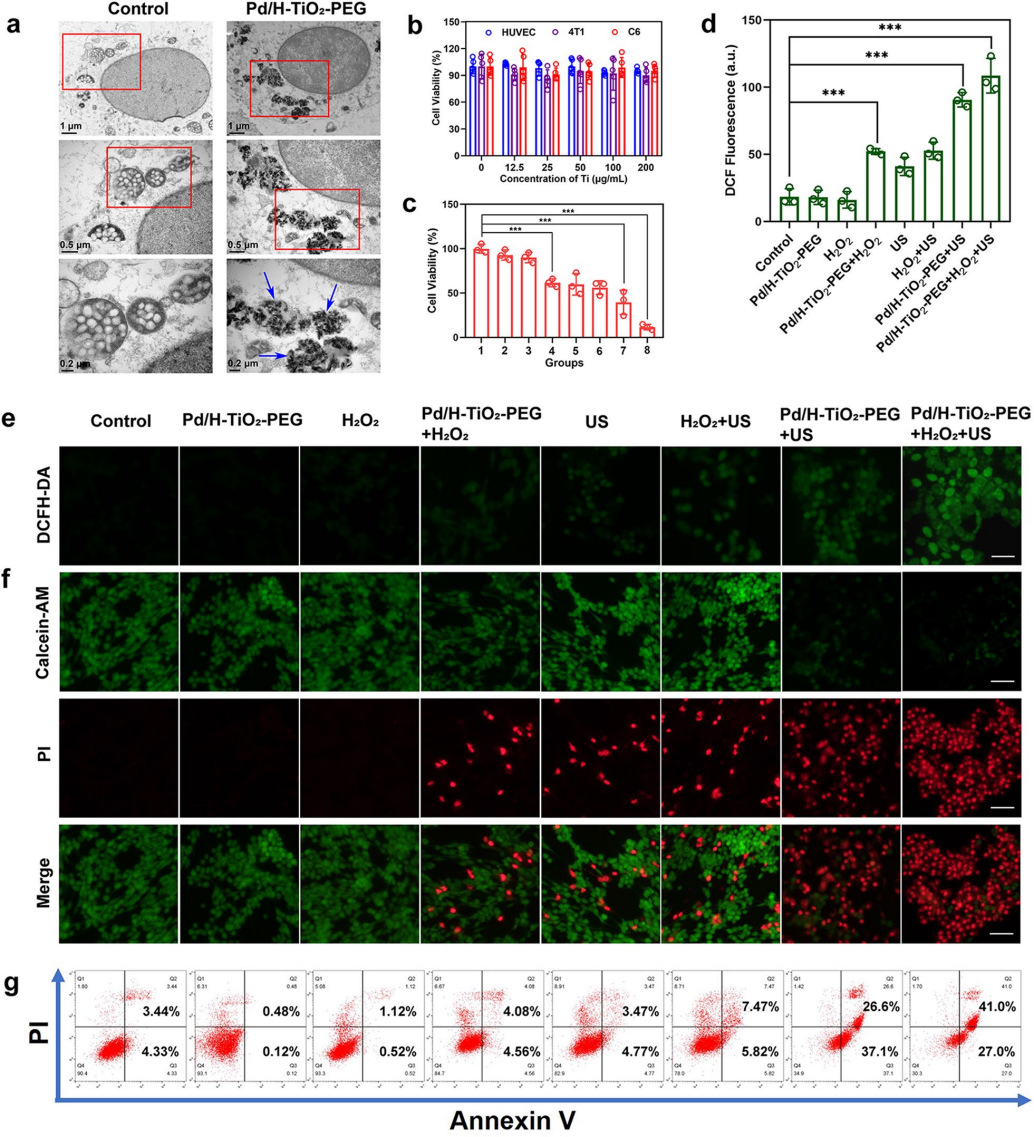
SDT and CDT effects of Pd/H-TiO_2_-PEG at the cellular level. **a** Bio-TEM images of C6 cells after incubation without and with Pd/H-TiO_2_-PEG. Scale bars: 1 μm, 0.5 μm and 0.2 μm successively. **b** Relative viabilities of HUVECs, 4T1 and C6 cells after incubation with elevated concentrations of Pd/H-TiO_2_-PEG. **c** Relative viabilities of C6 cells subjected to varied interventions: (1) Control, (2) Pd/H-TiO_2_-PEG, (3) H_2_O_2_, (4) Pd/H-TiO_2_-PEG + H_2_O_2_, (5) US, (6) H_2_O_2_ + US, (7) Pd/H-TiO_2_-PEG + US and (8) Pd/H-TiO_2_-PEG + H_2_O_2_ + US. **d** Quantitative fluorescent intensity of DCF in different groups. **e** Fluorescence images of C6 cells stained with DCFH-DA after suffering varied interventions. Scale bars: 25 μm. **f** Fluorescence images of C6 cells costained with Calcein-AM and PI after suffering varied interventions. Scale bars: 50 μm. **g** Flow cytometry apoptosis analysis of C6 cells costained with Annexin V and PI after being subjected to varied interventions

To reveal the antitumor potential of Pd/H-TiO_2_-PEG, the toxicity evaluation at the cellular level ensued. Certainly, affirmative intracellular uptake of Pd/H-TiO_2_-PEG was the precondition to ensure the effective killing to cancer cells. To acquire the evidence of endocytosis, cells were observed under the bio-TEM after coincubation without and with Pd/H-TiO_2_-PEG for 24 h. Encouragingly, pronounced clusters of Pd/H-TiO_2_-PEG (indicated by blue arrows) were easily found in the cytoplasm (Fig. 4a), affording a strong basis for Pd/H-TiO_2_-PEG to play its tumor killing role. Biocompatibility of nanoparticles was a vital indicator for future biological application. Here, the CCK-8 assay was used to assess the relative viabilities of human umbilical vein endothelial cells (HUVECs), 4T1 breast cancer cells and C6 glioma cells after cocultivation with increased doses (0, 12.5, 25, 50, 100, and 200 μg mL^−1^, based on Ti) of Pd/H-TiO_2_-PEG for 24 h. No matter for normal HUVECs, 4T1 or C6 cancer cells, Pd/H-TiO_2_-PEG alone showed no marked toxicity (Fig. 4b), reminding the innocuity of Pd/H-TiO_2_-PEG when lacking external incentives. However, when stimuli including H_2_O_2_, US and H_2_O_2_ + US were imposed after the coculture of C6 cells with different doses of Pd/H-TiO_2_-PEG, cell viabilities gradually declined with the elevated concentrations (Additional file 1: Figure S10) due to the generation of ROS through CDT and SDT. For further cytotoxicity investigation of SDT, CDT and SDT + CDT, cell viabilities were compared after experiencing diverse managements. As exhibited in Fig. 4c, cell viabilities significantly reduced in SDT group (group 7, Pd/H-TiO_2_-PEG + US) and CDT group (group 4, Pd/H-TiO_2_-PEG + H_2_O_2_), and the viability decline was even more in SDT + CDT group (group 8, Pd/H-TiO_2_-PEG + H_2_O_2_ + US), uncovering the synergetic enhancement of SDT and CDT.

ROS was the key medium for Pd/H-TiO_2_-PEG to assault cancer cells, therefore the intracellular ROS level was subsequently decided using the general probe DCFH-DA of which the hydrolysate in cells could be oxidized to fluorescent 2,7-dichlorofluorescein (DCF) by ROS. When cells were treated with Pd/H-TiO_2_-PEG + US, the strong green fluorescence was explicitly noticed, indicating a large deal of ROS production through SDT. The weaker fluorescence in Pd/H-TiO_2_-PEG + H_2_O_2_ group suggested lower ROS output through CDT than through SDT, but when US was combined, the fluorescence was conspicuously highlighted (Fig. 4d, e), certifying the boost of ROS generation by US and the collaborative effect of SDT and CDT. To present the cellular state after various disposals, the Calcein-AM and PI double staining was adopted to stain live and dead cells. The piles of dead cells (red) in Pd/H-TiO_2_-PEG + H_2_O_2_ + US group (Fig. 4f) visually reflected the effective cell killing effect of sono-chemodynamic therapy. The synergy of SDT and CDT was further confirmed by flow cytometry apoptosis analysis in the light of the Annexin V-FITC and PI costaining procedures (Fig. 4g).

### Transcriptome alteration induced by combinatorial SDT and CDT

**Fig. 5:**
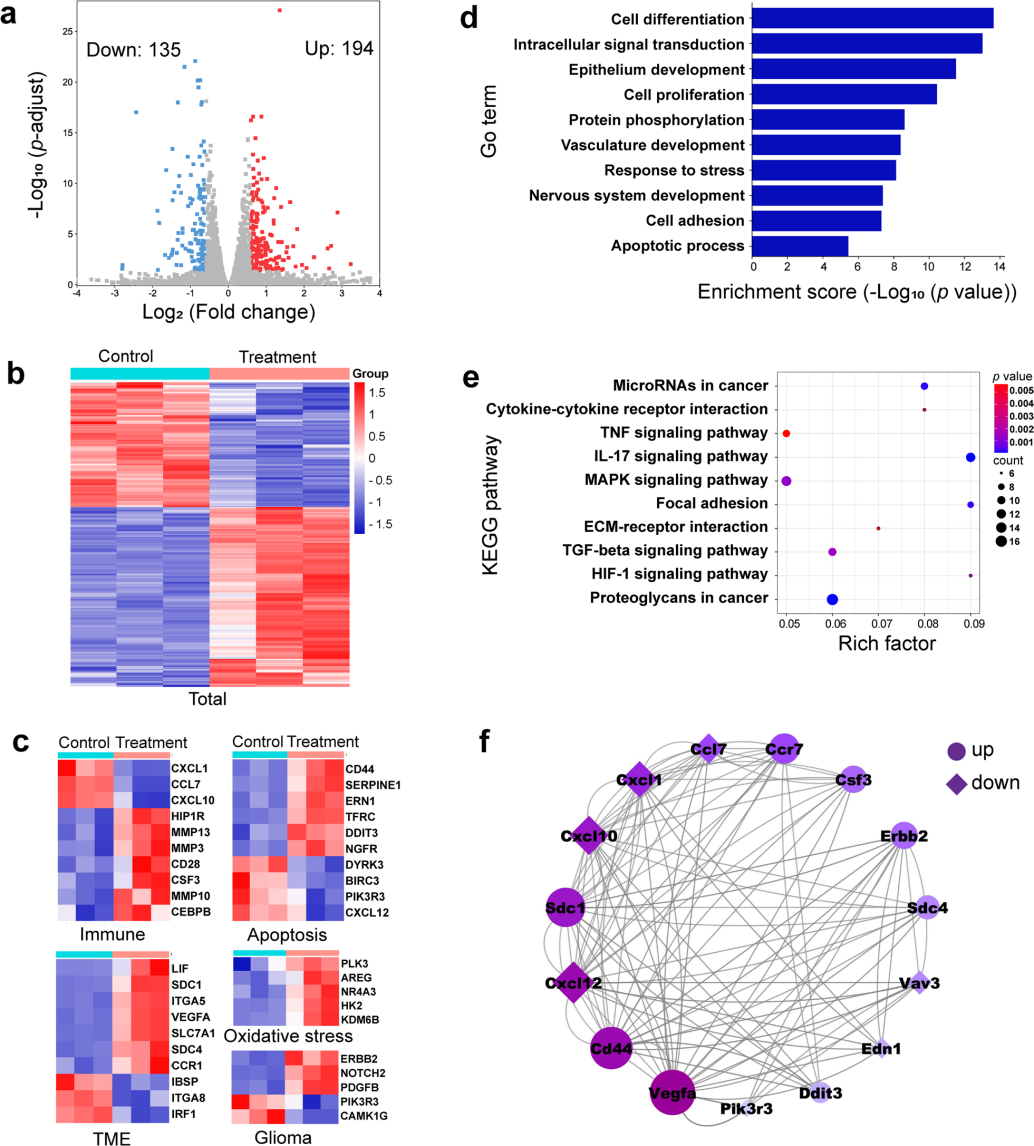
Gene expression analysis of SDT and CDT treated C6 cells by RNA sequencing. **a** Volcano map presenting the downregulated and upregulated genes in Pd/H-TiO_2_-PEG + H_2_O_2_ + US treated C6 cells (*p*-adjust < 0.05, absolute fold-change cut off ≥ 1.5). **b** Clustering analysis of DEGs in treated and untreated C6 cells. **c** Heatmaps of DEGs associated with immune, apoptosis, tumor microenvironment, oxidative stress and glioma. **d** GO and **e** KEGG enrichment analyses of functions and signal pathways that the related DEGs participated in. **f** PPI network of the proteins encoded by DEGs

To get a deep insight into the intrinsic mechanism of Pd/H-TiO_2_-PEG mediated combinatorial SDT and CDT therapy in vitro, RNA sequencing was performed to analyze the gene expression in C6 cells after being treated (treatment group) and not being treated (control group) with Pd/H-TiO_2_-PEG + H_2_O_2_ + US. A total of 329 differentially expressed genes (DEGs) were ascertained, with 135 downregulated genes (blue) and 194 upregulated genes (red) in the cells of treatment group compared with the control group based on the settings of *p*-adjust < 0.05 and absolute fold-change cut off ≥ 1.5 (Fig. 5a). The clustering analysis clearly screened and separated the total upregulated and down regulated genes in cells of the two groups (Fig. 5b). From Fig. 5c, we could see significant differences in the expression of genes correlated with immune, apoptosis and tumor microenvironment (TME), indicating that Pd/H-TiO_2_-PEG mediated combinatorial SDT and CDT could function through regulating chemokines (CXCL1, CCL7, CXCL10, etc.), immune response (HIP1R, CSF3, CD28, etc.), epithelial-mesenchymal transition (CD44, ITGA5, SDC4, etc.), and cell adhesion (SERPINE1, TFRC, IBSP, etc.). Specially, owing to the creation of ROS during treatment, oxidative stress related genes (PLK3, NR4A3, KDM6B, etc.) involved in DNA repair were upregulated, suggesting the damage of ROS to C6 cells. In addition, the treatment could specifically induce the alteration of glioma related genes (ERBB2, NOTCH2, PDGFB, etc).

Gene ontology (GO) enrichment analysis distinctly explicated that DEGs were mainly concentrated in cell differentiation, intracellular signal transduction, epithelium development, cell proliferation and protein phosphorylation (Fig. 5d), all of which were indispensable biological processes for tumorigenesis and progression. The result of Kyoto encyclopedia of genes and genomes (KEGG) enrichment illuminated that DEGs were mostly implicated in MicroRNAs in cancer, cytokine-cytokine receptor interaction, TNF signaling pathway, IL-17 signaling pathway and MAPK signaling pathway (Fig. 5e). Generally, the enrichment of DEGs in TNF and IL-17 signaling pathways supported that Pd/H-TiO_2_-PEG + H_2_O_2_ + US treatment promoted tumor cell death mainly through the mechanism of regulating cell apoptosis and antitumor immune response. Specially, it could be inferred from the enrichment of MAPK signaling pathway that ROS triggered oxidative stress was a powerful driver of cell apoptosis. In addition, the HIF-1 signaling pathway was enriched, directly affirming the crucial role of oxygen in cancer therapy. The protein–protein interaction (PPI) network elucidated the interconnection between the proteins encoded by DEGs (Fig. 5f), laying the foundation for the in-depth mechanism research of the treatment.

### In vivo biosafety, pharmacokinetics and biodistribution of Pd/H-TiO_2_-PEG

The biosafety of nanomedicines was a paramount prerequisite for in vivo application and future clinical translation. Therefore, the systemic toxicity of Pd/H-TiO_2_-PEG was assessed in priority. Briefly, after intravenous administration of different doses of Pd/H-TiO_2_-PEG on healthy mice, the body weight of each mouse was regularly recorded during 30 days, and blood indexes together with organ pathology were examined in the end. As shown in Additional file 1: Figure S11, body weights of the mice were constantly on the rise and there was no obvious discrepancy between all groups within the whole observation period. From the H&E staining images, we barely perceived any histological changes of major organs (heart, liver, spleen, lung and kidney) in the mice (Additional file 1: Figure S12). Also, the blood test results displayed that the haematological variables as well as the parameters of hepatic and renal functions were within normal limits and no relationship was presented between Pd/H-TiO_2_-PEG doses and the indexes (Additional file 1: Figure S13). The above results collectively illustrated the assured biosafety of Pd/H-TiO_2_-PEG, and this precondition laid the groundwork for the follow-up in vivo steps.

The pharmacokinetics and tissue distribution studies were conducive to disclosing the accumulative and metabolic processes of Pd/H-TiO_2_-PEG, being essential parts for guiding to establish an optimal treatment regimen. By detecting the Ti content of blood samplings collected at different time points post intravenous injection of Pd/H-TiO_2_-PEG, we got the blood circulation and eliminating rate curves of Pd/H-TiO_2_-PEG, which precisely told that Pd/H-TiO_2_-PEG had a relatively long blood half-life of 3.54 h and the eliminating rate descended from 0.39 μg mL^−1^ per h to 0.02 μg mL^−1^ per h at 1.36 h (Additional file 1: Figure S14). Thereupon, the biodistribution of Pd/H-TiO_2_-PEG in major organs and the tumor site was investigated on C6 tumor-bearing mice. It turned out that Pd/H-TiO_2_-PEG could overtly enrich in the tumor at 24 h post injection, contributing to deciding the rational interval between the medication and US irradiation (Additional file 1: Figure S15).

### Pd/H-TiO_2_-PEG NSs mediated in vivo tumor inhibition

**Fig. 6:**
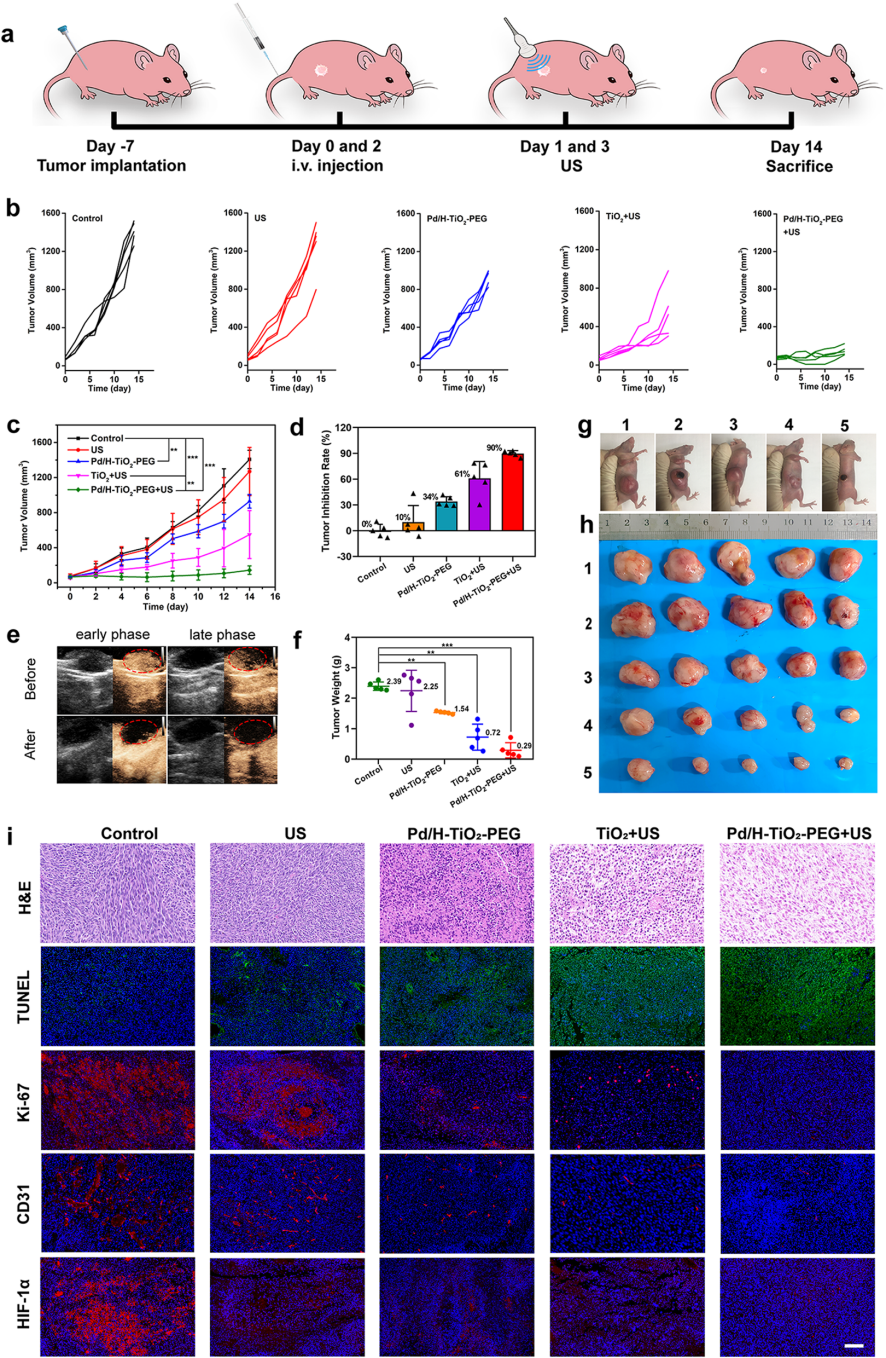
In vivo therapeutic performance of Pd/H-TiO_2_-PEG against C6 tumor in the mouse. **a** Therapeutic regimen for C6 tumor-bearing mice. Growth curves of **b** individual tumor and **c** each group of tumors. **d** Tumor inhibition rate of each group. **e** CEUS images of the tumor before and after Pd/H-TiO_2_-PEG + US treatment. **f** Tumor weights of the mice in all groups at the endpoint. **g** C6 tumor-bearing mice and **h** resected tumors in each group at the terminal of treatment. (1) Control, (2) US, (3) Pd/H-TiO_2_-PEG, (4) TiO_2_ + US and (5) Pd/H-TiO_2_-PEG + US. **i** H&E, TUNEL, Ki-67, CD31 and HIF-1α staining of the tumor tissues suffered various treatments. Scale bars:100 μm

The satisfying in vitro therapeutic efficacy and comprehensive in vivo pre-work massively propelled the exploration of the in vivo antitumor action of Pd/H-TiO_2_-PEG. The BALB/c nude mice bearing subcutaneous C6 tumors were randomly divided into five groups (n = 5 in each group): (1) Control (PBS), (2) US, (3) Pd/H-TiO_2_-PEG, (4) TiO_2_ + US and (5) Pd/H-TiO_2_-PEG + US. The dosage of TiO_2_ and Pd/H-TiO_2_-PEG was the same as 100 μL (10 mg kg^−1^, *i.v.* injection) and the US settings were 1.5 W cm^−2^, 1.0 MHz, 50% duty cycle and 3 min. The mice in group 1, 3, 4 and 5 firstly underwent intravenous administration of varied reagents and then the mice in all groups were treated with or without US at 24 h post injection. The same protocols were executed on the second day and the whole observation period was 14 days (Fig. 6a), during which the body weight and tumor dimensions of each mouse were monitored every other day. The growth curves of individual tumor (Fig. 6b) and each group of tumors (Fig. 6c) manifested affirmative tumor restraint in Pd/H-TiO_2_-PEG group (CDT), TiO_2_ + US group (SDT) and especially in Pd/H-TiO_2_-PEG + US group (CDT + SDT), with the tumor inhibition rate of 34%, 61% and 90%, respectively (Fig. 6d), while body weights of the mice steadily increased with time (Additional file 1: Figure S16). The results corroborated the indisputable antitumor efficacy of bilaterally enhanced SDT and CDT with neglectable side effects. The CEUS images of the tumor before and after Pd/H-TiO_2_-PEG + US treatment revealed evident reduction of the internal blood supply (Fig. 6e), confirming the destruction of tumor vasculature. Furthermore, at the end of the observation period, the lightest weight and minimum size of the tumors in Pd/H-TiO_2_-PEG + US group also offered convincing proofs for the therapeutic effect of synergistic SDT and CDT (Fig. 6f-h).

Ultimately, tumor tissues in all groups underwent H&E, TUNEL, Ki-67, CD31 and HIF-1α staining for exhaustive histological analyses, and the pathological changes could be straightly identified from Fig. 6i and Additional file 1: Figure S17. From H&E and TUNEL staining images we could easily observe the chromatin condensation, nuclear disintegration and strongest green fluorescence signal in Pd/H-TiO_2_-PEG + US group, attesting that Pd/H-TiO_2_-PEG + US disposal brought about severe cell apoptosis. The significantly decreased red fluorescence signal of Ki-67 and CD31 suggested the inhibition of cell proliferation and tumor angiogenesis. The eximious oxygen supply ability of Pd/H-TiO_2_ has been verified in vitro. Here, the in vivo hypoxia remission capability was testified by detecting HIF-1α, a key factor that highly expressed in hypoxia tissues to regulate cells’ adaptive responses to low oxygen tension. From Fig. 6i, we could see the most conspicuous red fluorescence signal of HIF-1α in control group, reflecting severe tissue hypoxia. In comparison, the hypoxic condition was significantly alleviated in Pd/H-TiO_2_-PEG and Pd/H-TiO_2_-PEG + US groups thanks to the catalytic action of Pd component. Noteworthily, in the TiO_2_ + US group, the expression of HIF-1α was also decreased compared with the control group, which had nothing to do with Pd. For this, it was deemed that the hypoxia amelioration was credited to the reduced oxygen consumption following tumor shrinkage caused by TiO_2_ + US induced SDT effect. Except for the histological proof, the enhanced CEUS signal after intratumorally injection of Pd/H-TiO_2_-PEG also intuitively testified its intratumoral oxygen generation property (Additional file 1: Figure S18), which was crucial assistance for the reinforcement of SDT and CDT. Besides, no visible pathological damage of major organs in all groups was perceived through H&E staining (Additional file 1: Figure S19), implying the indubitable therapeutic safety. On the whole, Pd/H-TiO_2_-PEG NSs could be used as innocuous and reliable sonosensitizers with integrated SDT, CDT and oxygen production effects to achieve a satisfactory therapeutic efficiency for cancers.

## Conclusions

In summary, 2D black Pd/H-TiO_2_ composite nanosonosensitizers with oxygen deficiencies and surface disorder for high-efficiency cancer assault have been designed and engineered by using a gentle hydrogenation approach. Apparently, these Pd/H-TiO_2_ NSs exhibit efficient US-evoked ROS generation compared with conventional TiO_2_ NSs, which is ascribed to the reconstruction of the top two layers of TiO_2_ surface and the resultant narrowed bandgap as supported by DFT calculations. In the meantime, the presence of Ti^3+^ can react with the tumor endogenous H_2_O_2_ under the acidic physiological microenvironment to form •OH, this boosts ROS to a higher level by combining with SDT-evoked ROS, and the synergy of CDT and SDT for robust cancer killing is systematically accomplished. Furthermore, hypoxia, the hard nut to crack facing cancer therapy, is simultaneously conquered, for the incorporated Pd nanoparticles located on TiO_2_ substrates during the black TiO_2_ construction play the role of catalase which can turn the nanoplatform into an oxygen manufactory. The three-pronged trait harbored by Pd/H-TiO_2_ NSs makes them excel at oncotherapy, which has been attested by ROS-induced cell death in vitro, indubitable tumor suppression in vivo as well as the significant transcriptome alteration detected by RNA sequencing. This work not only opens up a span-new window for the simplified design of versatile TiO_2_-based nanocomposites with outstanding SDT-enabled tumor inhibition rate, but also sparks the inspirations of contriving potent metal oxide-based nanomedicines for the worldwide battle against cancer.

## Supplementary Information

**Additional file 1.** Additional information includes part of materials and methods, as well as additional figures.

## Data Availability

The datasets and materials used in the study are available from the corresponding author.
