# Supplementary material for: Engineering defected 2D Pd/H-TiO2 nanosonosensitizers for hypoxia alleviation and enhanced sono-chemodynamic cancer nanotherapy
Source: J Nanobiotechnology. 2022 Apr 12;20:186. doi: 10.1186/s12951-022-01398-6 (PMC9003983; doi:10.1186/s12951-022-01398-6)
Supplement: Supplementary file 1 — Additional file 1. Additional information includes part of materials and methods, as well as additional figures. [file 12951_2022_1398_MOESM1_ESM.docx]

**Additional file 1:**

**Engineering Defected 2D Pd/H-TiO_2_ Nanosonosensitizers for Hypoxia Alleviation and Enhanced Sono-Chemodynamic Cancer Nanotherapy**

Xiaohui Qiao^1†^, Liyun Xue^1†^, Hui Huang^2^, Xinyue Dai^2*^, Yu Chen^2*^ and Hong Ding^1*^

^*^Correspondence: [daixinyuesdu@hotmail.com](mailto:daixinyuesdu@hotmail.com) (X. Dai); [chenyuedu@shu.edu.cn](mailto:chenyuedu@shu.edu.cn) (Y. Chen); [ding_hong@fudan.edu.cn](mailto:ding_hong@fudan.edu.cn) (H. Ding)

^†^Xiaohui Qiao and Liyun Xue contributed equally to this work

^1^ Department of Ultrasound, Huashan Hospital, Fudan University, Shanghai, 200040, P. R. China.

^2^ Shanghai Engineering Research Center of Organ Repair, Materdicine Lab, School of Life Sciences, Shanghai University, Shanghai, 200444, P. R. China.

**Additional details on experimental section**

Materials

Tetrabutyl titanate (TBT, 99%), sodium tetrachloropalladate (II) (Na_2_PdCl_4_, 98%), 2,2,6,6-tetramethylpiperidine (TEMP, 98%), ethylene glycol (99%) and polyvinylpyrrolidone (PVP, MW 40000) were purchased from Chengdu Huaxia Chemical Reagent Co., Ltd. Dimethyl sulfoxide (DMSO, 99%), 1,3-diphenylisobenzofuran (DPBF, 97%), 2′,7′-dichlorofluorescin diacetate (DCFH-DA, 97%), methylene blue (MB, 96%), hydrogen peroxide (H_2_O_2_, 30 wt%), hydrofluoric acid (HF, 40%) and ethanol were obtained from Sigma-Aldrich. Amino-polyethylene glycol (NH_2_-PEG_2000_) was provided by Xi'an Ruixi Biotechnology Co., Ltd. 5,5-dimethyl-1-pyrroline N-oxide (DMPO, 97%) was purchased from Dojindo Molecular Technologies, Inc.

Calculation Method

Density functional theory calculations with the Hubbard correction (DFT+U) were performed to explore the geometric configurations of H adsorbed on the TiO_2_(001) surface. The strong-correlated correction was considered with generalized gradient approximation (GGA) +U method to deal with the Ti-3d electrons and the estimated U value for Ti-3d orbitals was set to be 7.8 eV [1-2] here and had been confirmed in bulk anatase TiO_2_ (**Fig. S1**), which had a bandgap of 3.4 eV [3-5]. All the DFT calculations were carried out by the Vienna Ab-initio Simulation Package (VASP) [6] with the van der Waals (vdW) interactions in terms of semi-empirical DFT-D3 correction [7]. The exchange-correlation function was the GGA of Perdew-Burke-Ernzerhof (PBE) [8] and the electronic plane wave interception energy was set to be 350 eV. A vacuum layer larger than 10 Å was used in modeling to avoid interactions between the neighboring periodic images. In structure optimizations, the energy convergence criteria of 10^-4^ eV and force convergence criteria of 0.01 eV/Å were used. For the bulk model with 4 pairs of TiO_2_, a 9×9×5 *k*-point Gamma mesh was chosen. While for the TiO_2_(001) and TiO_2_(001)-2H models with 45 pairs of TiO_2_, 3×3×1 *k*-point Gamma mesh was used to ensure adequate convergence.

Characterization

The hydrodynamic diameter of the nanoparticle was determined by dynamic light scattering (DLS, Zetasizer Nano ZS90, Malvern, UK). The transmission electron microscope (TEM), high-resolution transmission electron microscope (HRTEM), mapping plus energy dispersive spectrometer (EDS) analysis results were harvested by JEM 2100F (JEOL, Japan) to confirm the particle morphology, elemental varieties and relative contents. The scanning electron microscope (SEM) was completed on SU8020 (Hitachi, Japan) to reveal the surface morphology. The thickness of the nanoparticle was analyzed by atomic force microscope (AFM, Dimension Icon, Bruker, Germany). The crystalline structure was characterized by X-ray diffraction (XRD, D8 Advanced, Bruker, Germany). The surface composition, chemical states and valence band spectra were investigated by X-ray photoelectron spectroscopy (XPS, ESCALAB 250Xi, Thermo, USA). The ultraviolet-visible-near infrared (UV−vis−NIR) absorbance spectra were recorded by UV-1900i spectrophotometer (Shimadzu, Japan). The concentration of Ti was measured by inductively coupled plasma-mass spectrometer (ICP-MS, ICP-MS7800, Agilent, USA) and inductively coupled plasma-optical emission spectrometer (ICP-OES, ICP-OES730, Agilent, USA).

Cell Culture

The human umbilical vein endothelial cells (HUVECs), 4T1 breast cancer cells and C6 glioma cells were purchased from Cell Bank, Chinese Academy of Sciences (Shanghai) and cultured by Dulbecco’s modified eagle medium (DMEM) containing 10% fetal bovine serum and 1% penicillin/streptomycin.

Cellular Uptake

C6 cells were incubated with Pd/H-TiO_2_-PEG (100 μg mL^-1^) in a T 25 cell culture flask for 24 h. Then cells were collected, fixed, embedded and sliced for bio-TEM examination. In the meantime, cells without incubation with Pd/H-TiO_2_-PEG were collected as the comparison.

Cytotoxicity Assay

HUVECs, 4T1 cells and C6 cells were selected for cytotoxicity evaluation. Briefly, cells were seeded in 96-well plates at 1×10^4^ cells per well overnight, then the culture medium was replaced by the one containing increasing concentrations of Pd/H-TiO_2_-PEG (0, 12.5, 25, 50, 100, and 200 μg mL^-1^) and cells were continuously incubated for 24 h. The cell viability was evaluated by Cell Counting Kit-8 (CCK-8, Dojindo Molecular Technologies, Inc.) assay in the light of the standard protocol.

*In Vivo* Biocompatibility

Twenty female ICR mice were equally divided into four groups (n = 5 in each group) at random and injected with different concentrations of Pd/H-TiO_2_-PEG (0, 5, 10, and 15 mg kg^-1^, 100 μL) through the tail vein. The mice were weighed every other day for 30 days and then sacrificed. The blood samples were collected from the ocular veins for hemogram and biochemical indexes analyses, and the major organs including heart, liver, spleen, lung, and kidney were excised for H&E staining.

*In Vivo* Pharmacokinetics

Four female ICR mice were intravenously injected with 100 μL of Pd/H-TiO_2_-PEG (15 mg kg^-1^). 20 μL of blood was collected at 3 min, 5 min, 15 min, 30 min, 1 h, 2 h, 4 h, 8 h, and 24 h after injection for ICP test to measure the Ti content.

**Additional figures**


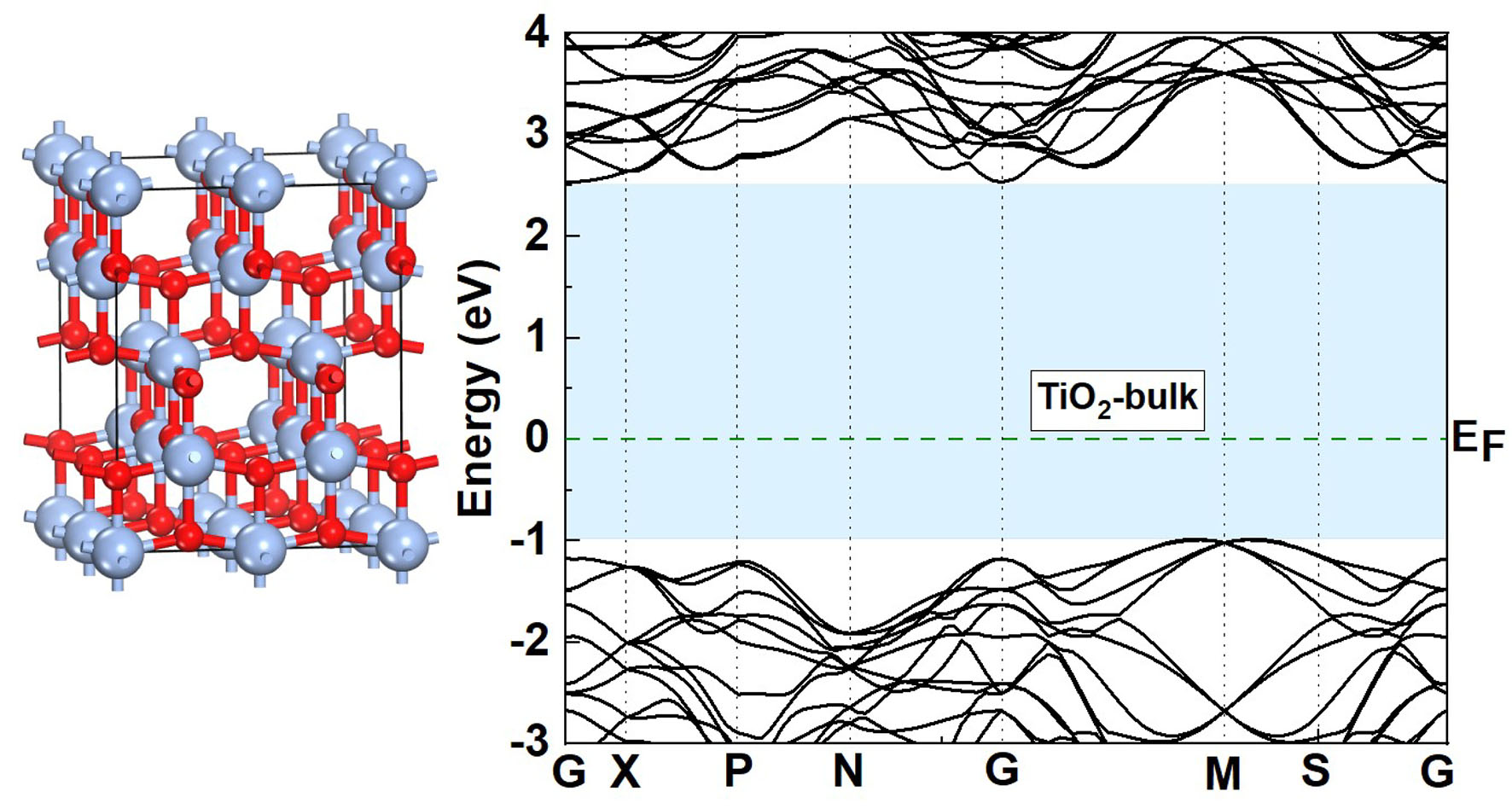


**Fig. S1.** Schematic and electronic band structure of bulk anatase TiO_2_ by GGA+U with U of 7.8 eV. G (0, 0, 0), X (0, 0, 0.5), P (0.25, 0.25, 0.25), N (0, 0.5, 0), M (0.5, 0.5, -0.5) and S (0.29, 0.71, -0.29), were the high-symmetric *k*-points in the first Brillouin-zone.


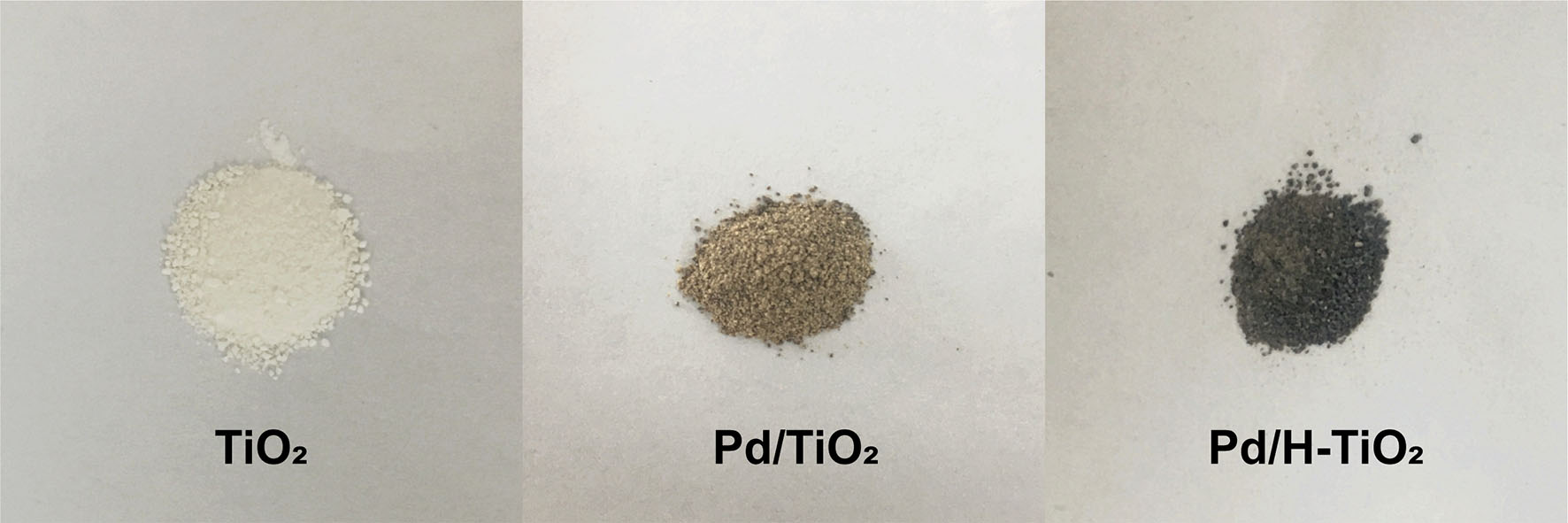


**Fig. S2.** Photographs of pure TiO_2_, Pd/TiO_2_ and Pd/H-TiO_2_.


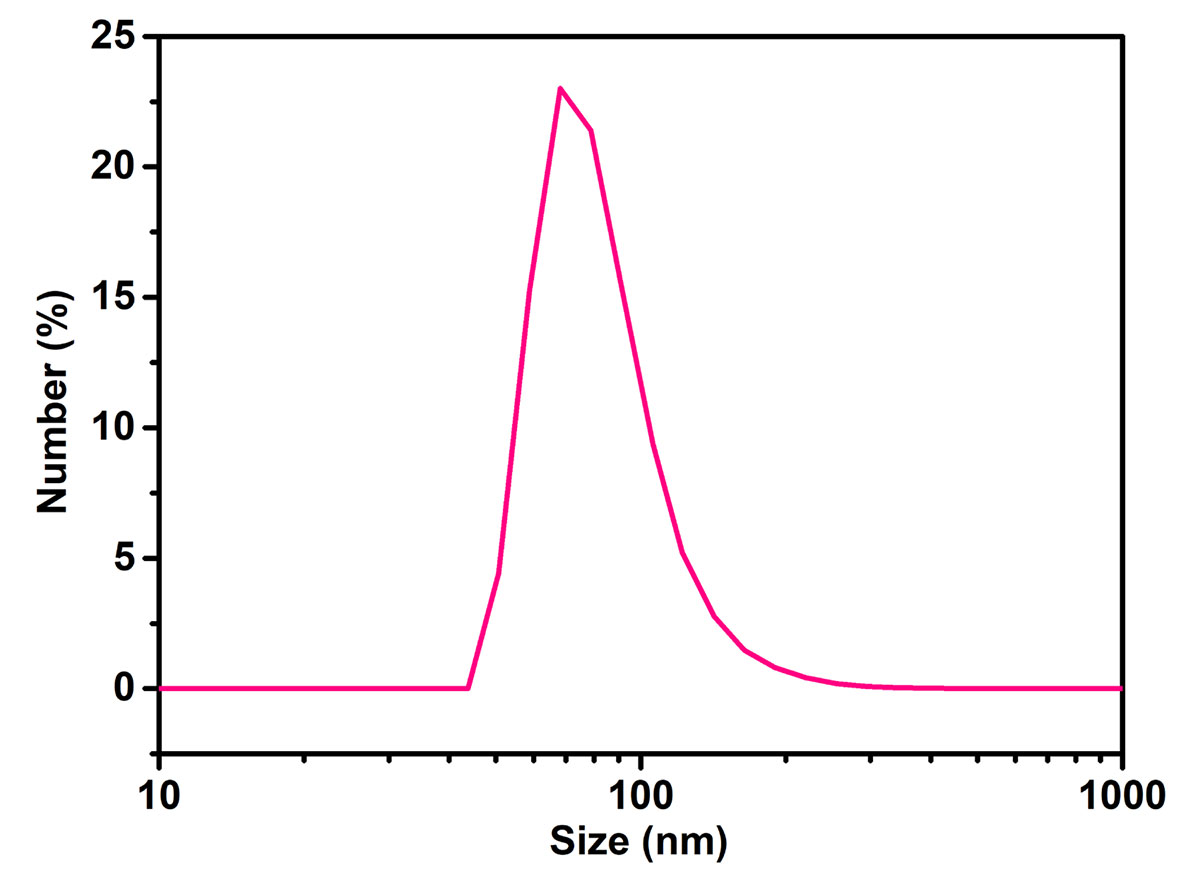


**Fig. S3.** Hydrodynamic diameter of Pd/H-TiO_2_-PEG measured by DLS.


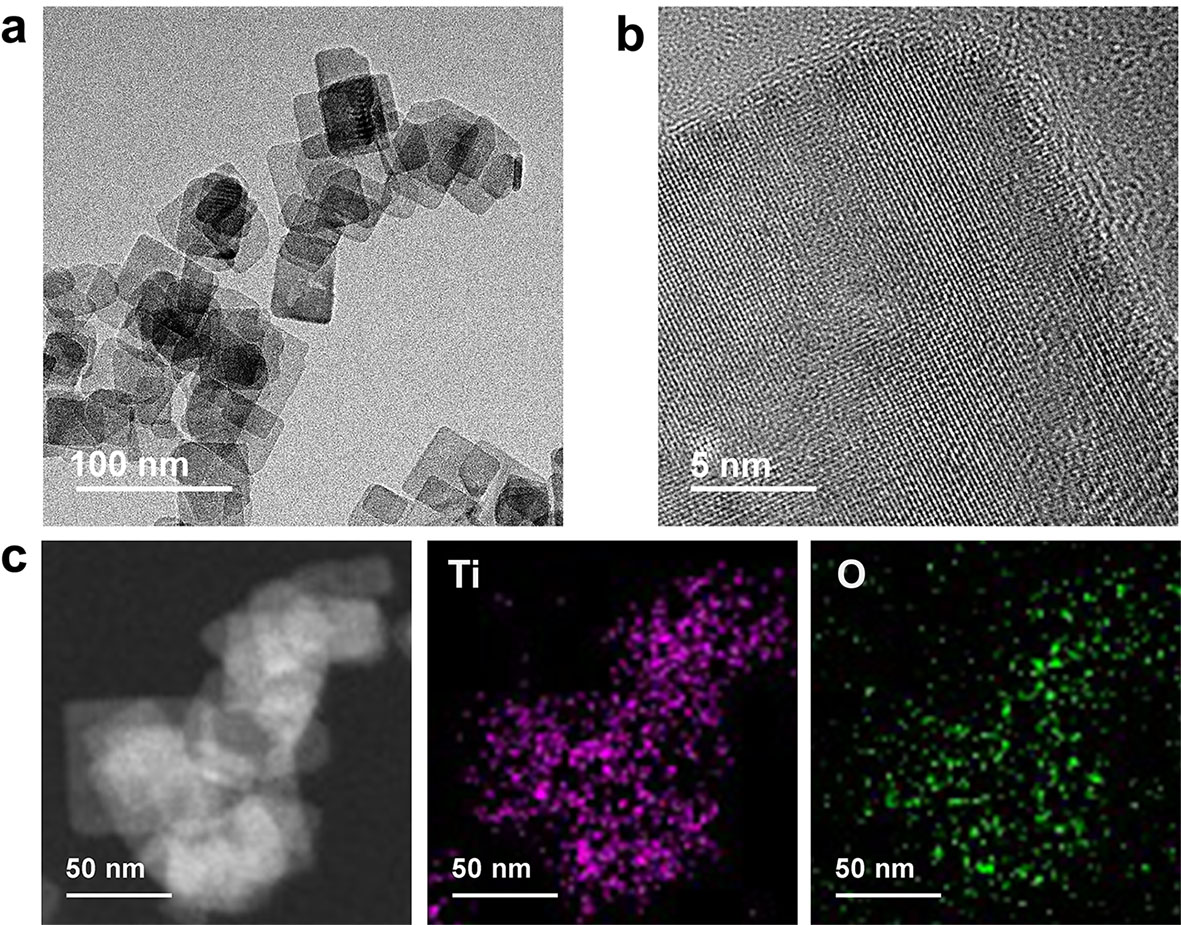


**Fig. S4.** TEM images of 2D TiO_2_ nanosheets. a) TEM and b) HRTEM images of TiO_2_. c) Elemental analysis of Ti and O in TiO_2_ nanosheets.


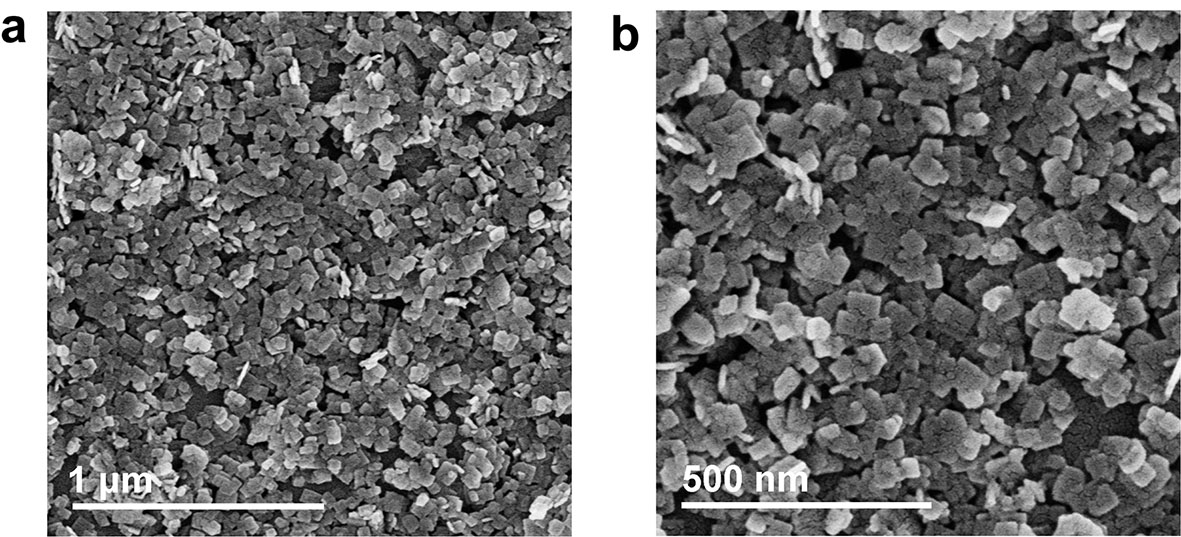


**Fig. S5.** SEM images of TiO_2_ nanosheets at varied magnifications.


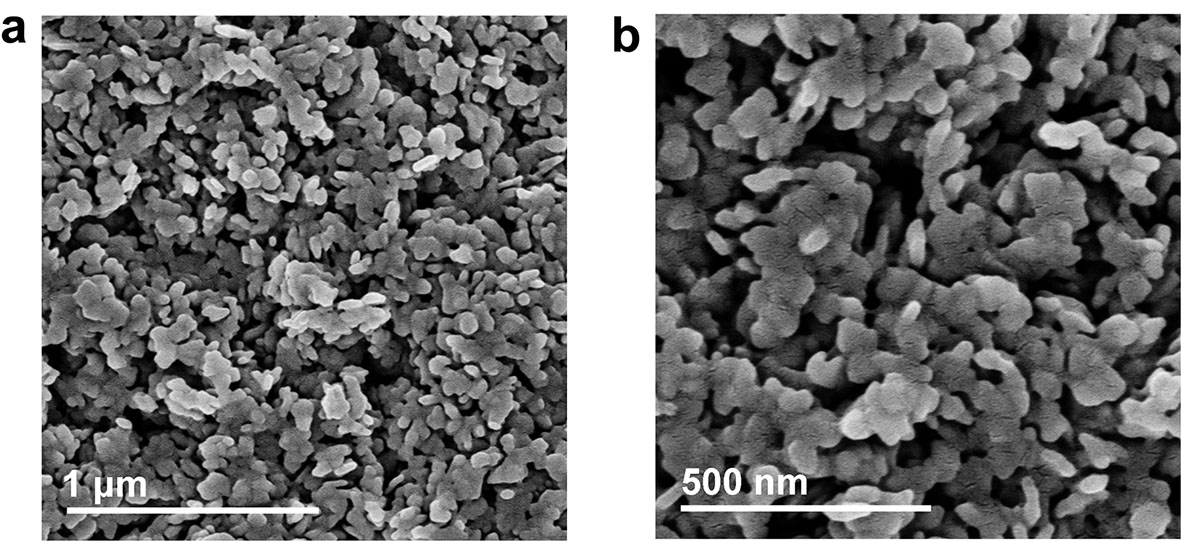


**Fig. S6.** SEM images of Pd/H-TiO_2_ nanosheets at different magnifications.


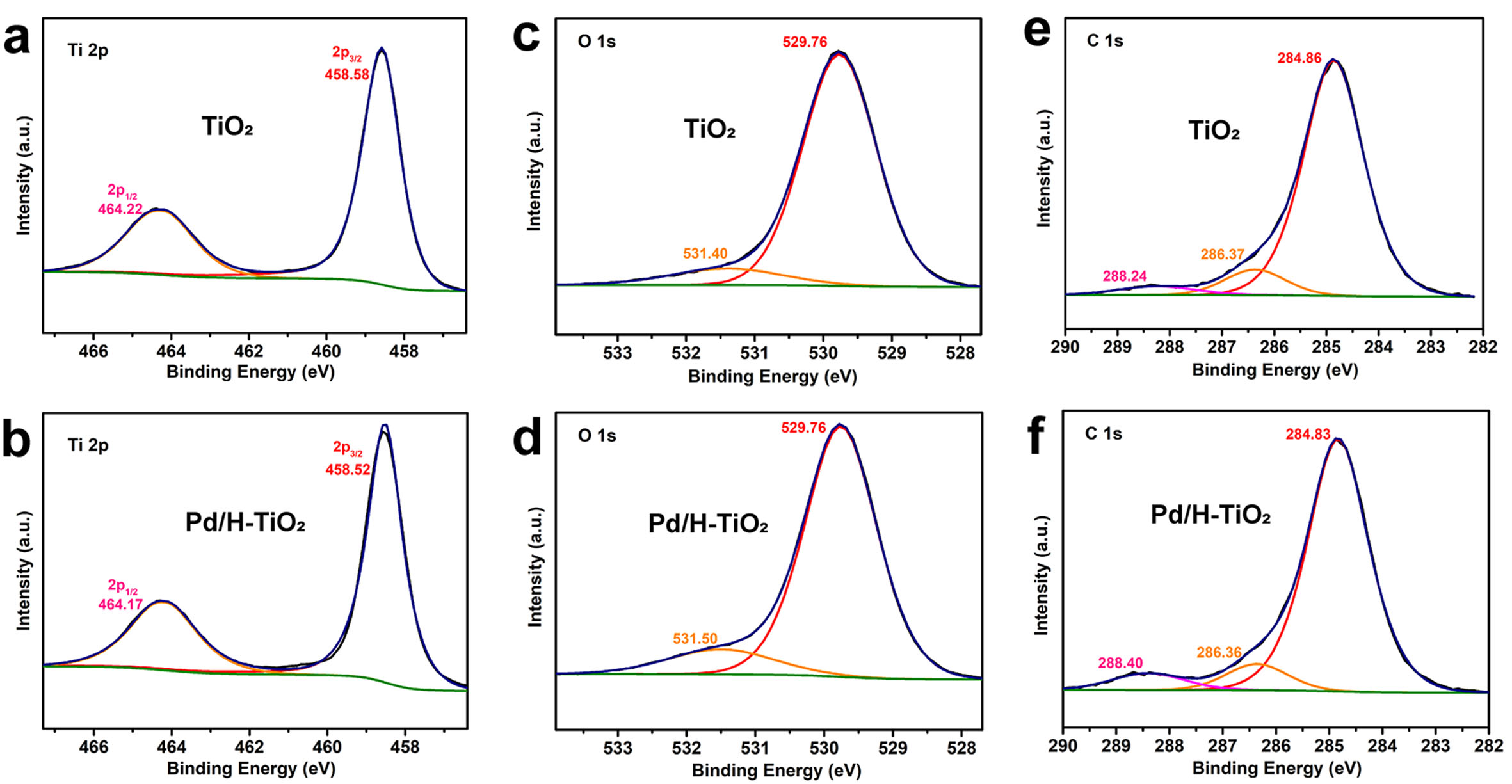


**Fig. S7.** XPS spectra of Ti 2p in a) TiO_2_ and b) Pd/H-TiO_2_. XPS spectra of O 1s in c) TiO_2_ and d) Pd/H-TiO_2_. XPS spectra of C 1s in e) TiO_2_ and f) Pd/H-TiO_2_.


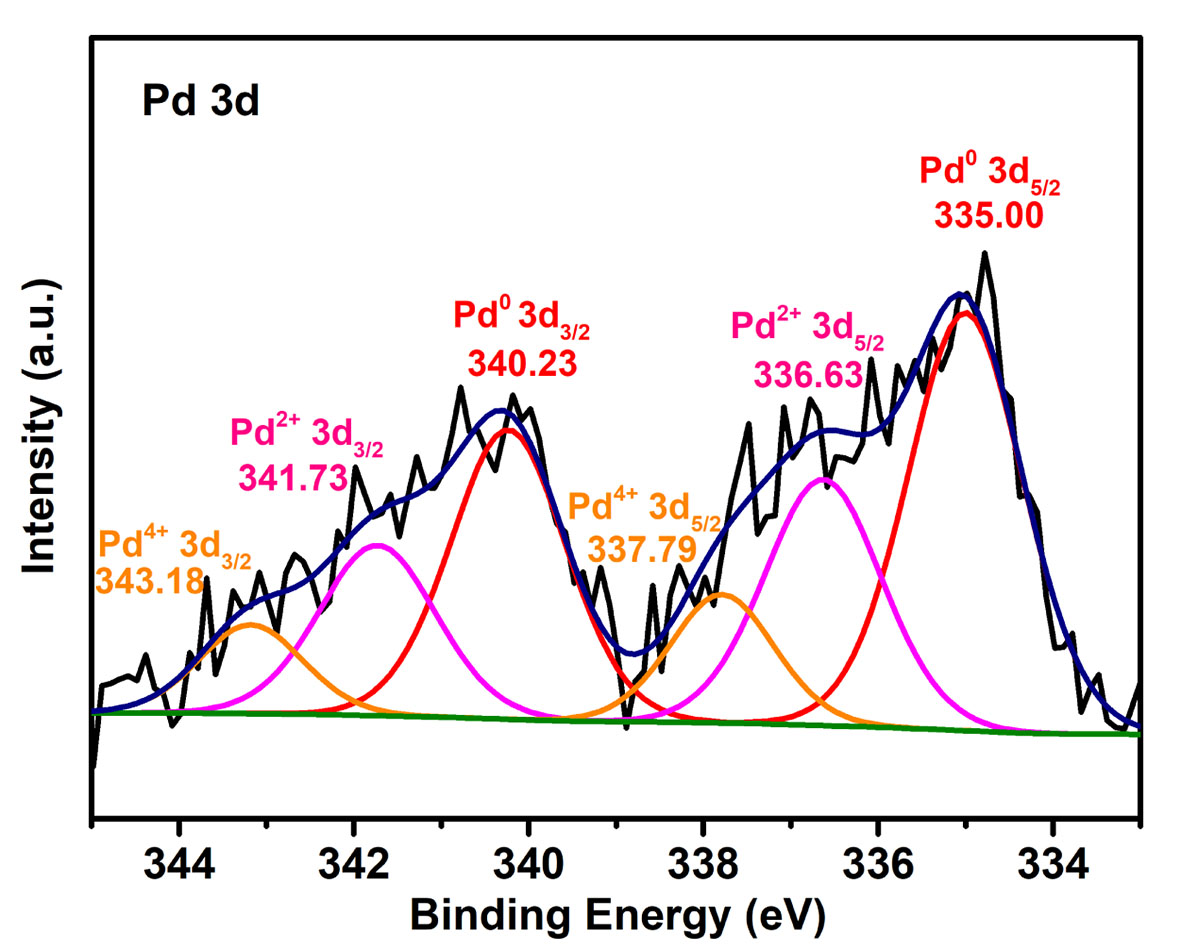


**Fig. S8.** XPS spectrum of Pd 3d in Pd/H-TiO_2_.


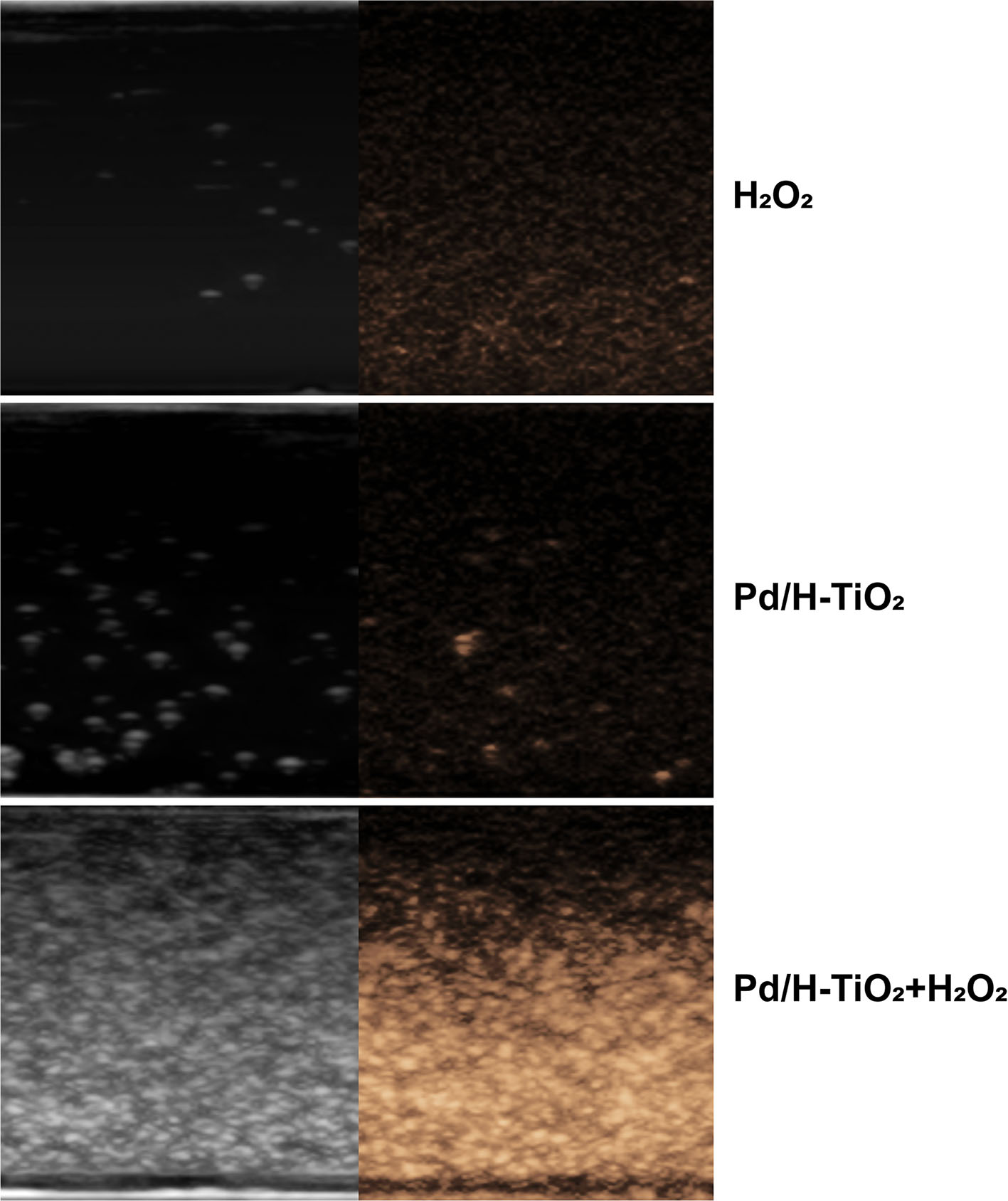


**Fig. S9.** *In vitro* oxygen generation from H_2_O_2_, Pd/H-TiO_2_ and Pd/H-TiO_2_ + H_2_O_2_ solutions observed by CEUS imaging.


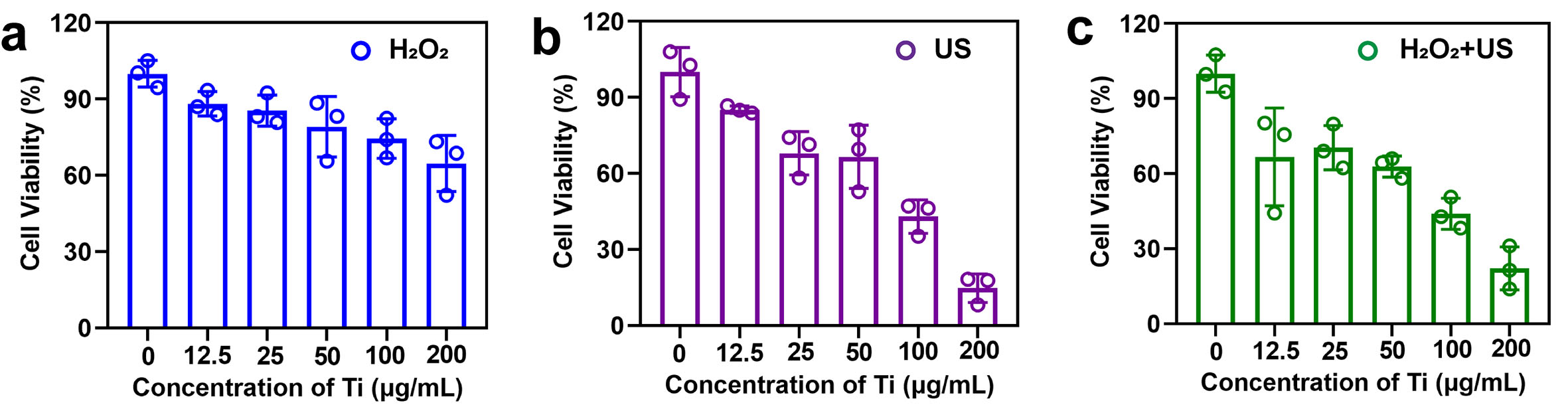


**Fig. S10.** Relative viabilities of C6 cells treated with H_2_O_2_, US and H_2_O_2_ + US after incubation with elevated concentrations of Pd/H-TiO_2_-PEG.


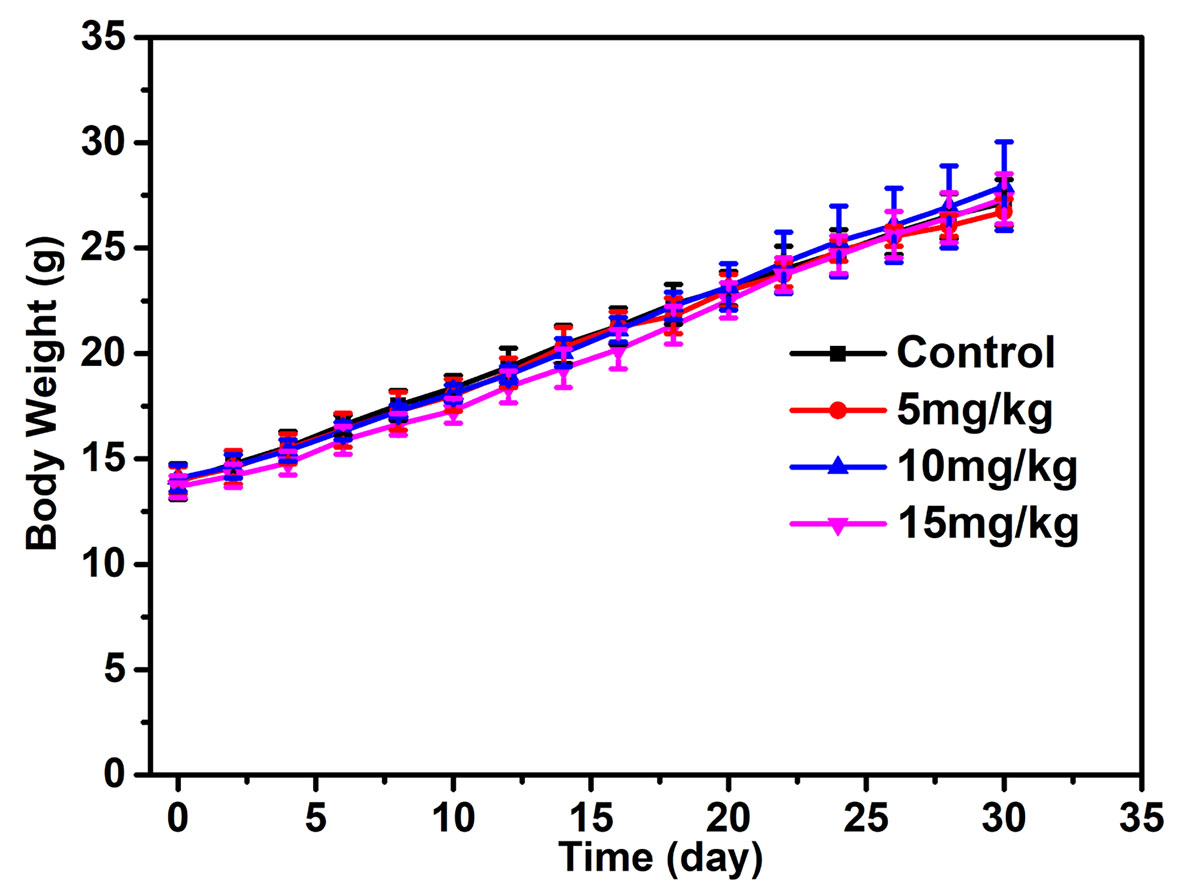


**Fig. S11.** Body weight curves of the mice treated with different concentrations of Pd/H-TiO_2_-PEG within 30 days.


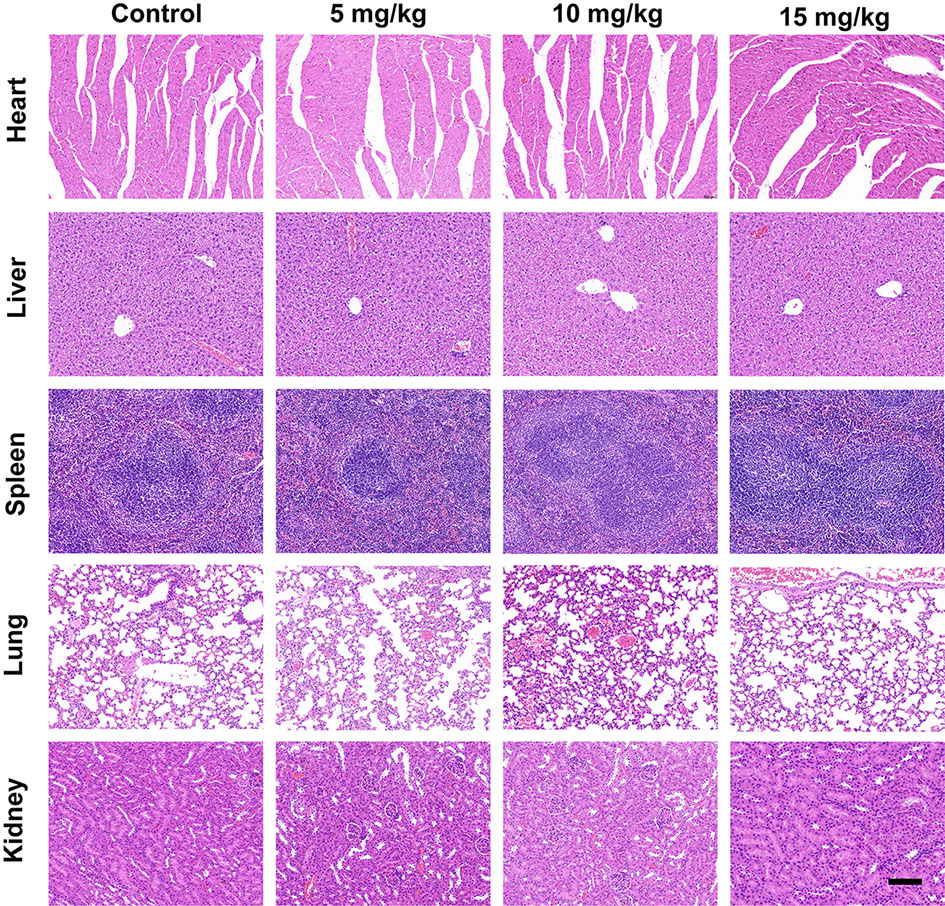


**Fig. S12.** H&E staining images of the major organs in the mice treated with different concentrations of Pd/H-TiO_2_-PEG for 30 days. Scale bars: 100 μm.


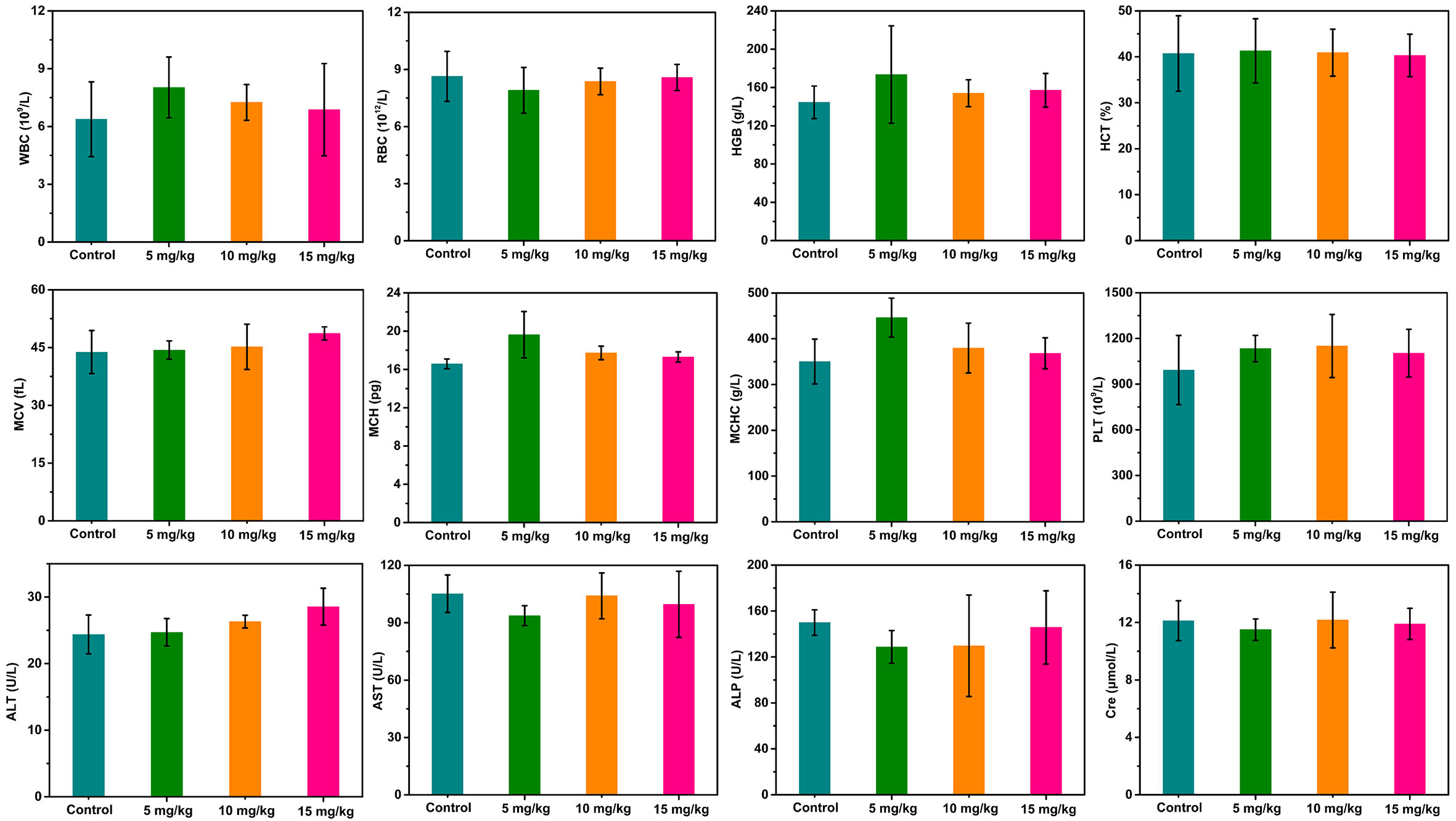


**Fig. S13.** Blood routine and biochemical indexes of the mice treated with different concentrations of Pd/H-TiO_2_-PEG for 30 days.


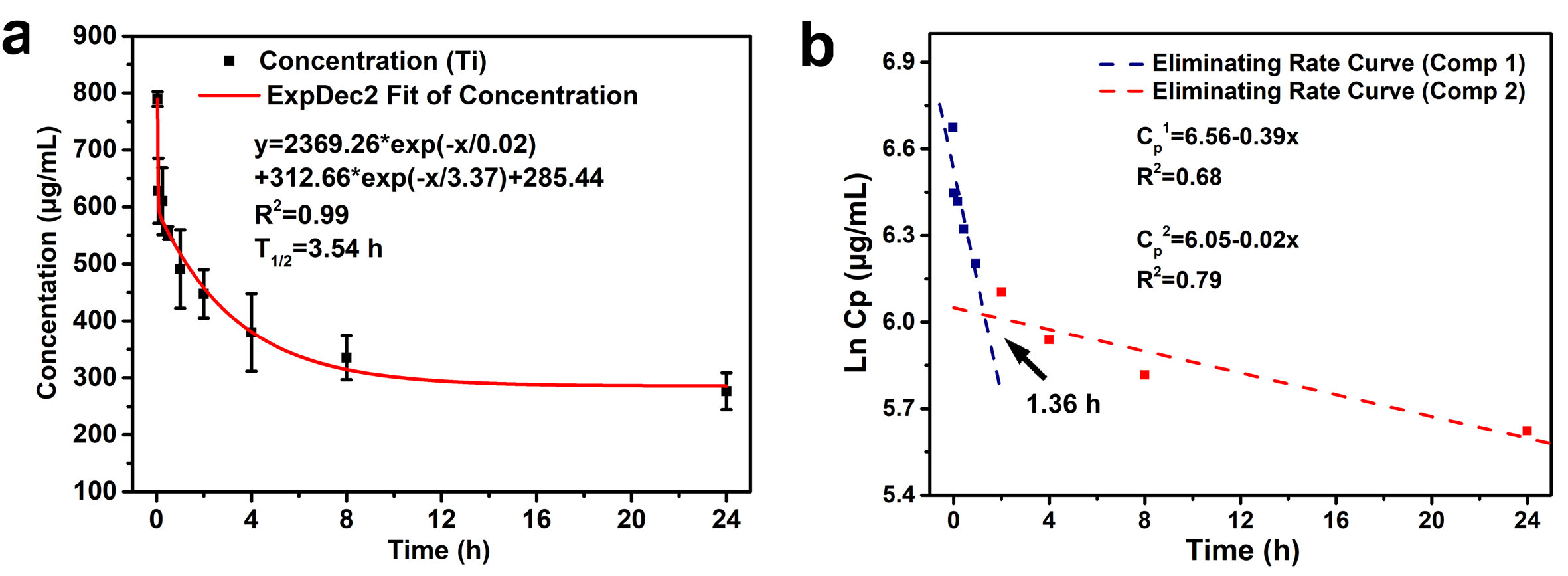


**Fig. S14.** *In vivo* pharmacokinetics of Pd/H-TiO_2_-PEG. a) The blood circulation curve of intravenously injected Pd/H-TiO_2_-PEG. b) The eliminating rate curve of Pd/H-TiO_2_-PEG.


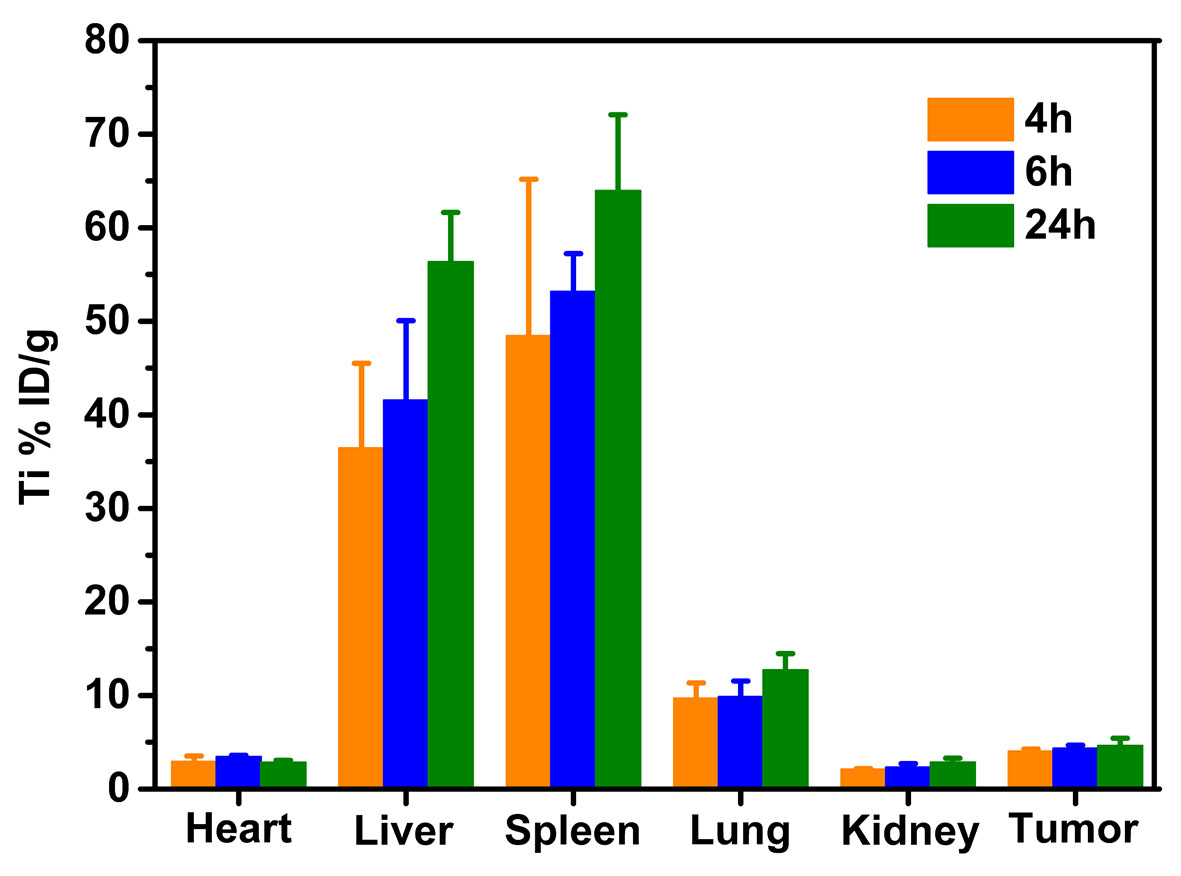


**Fig. S15.** Biodistribution of Ti in major organs and the tumor site at 4 h, 6 h and 24 h after intravenous injection of Pd/H-TiO_2_-PEG.


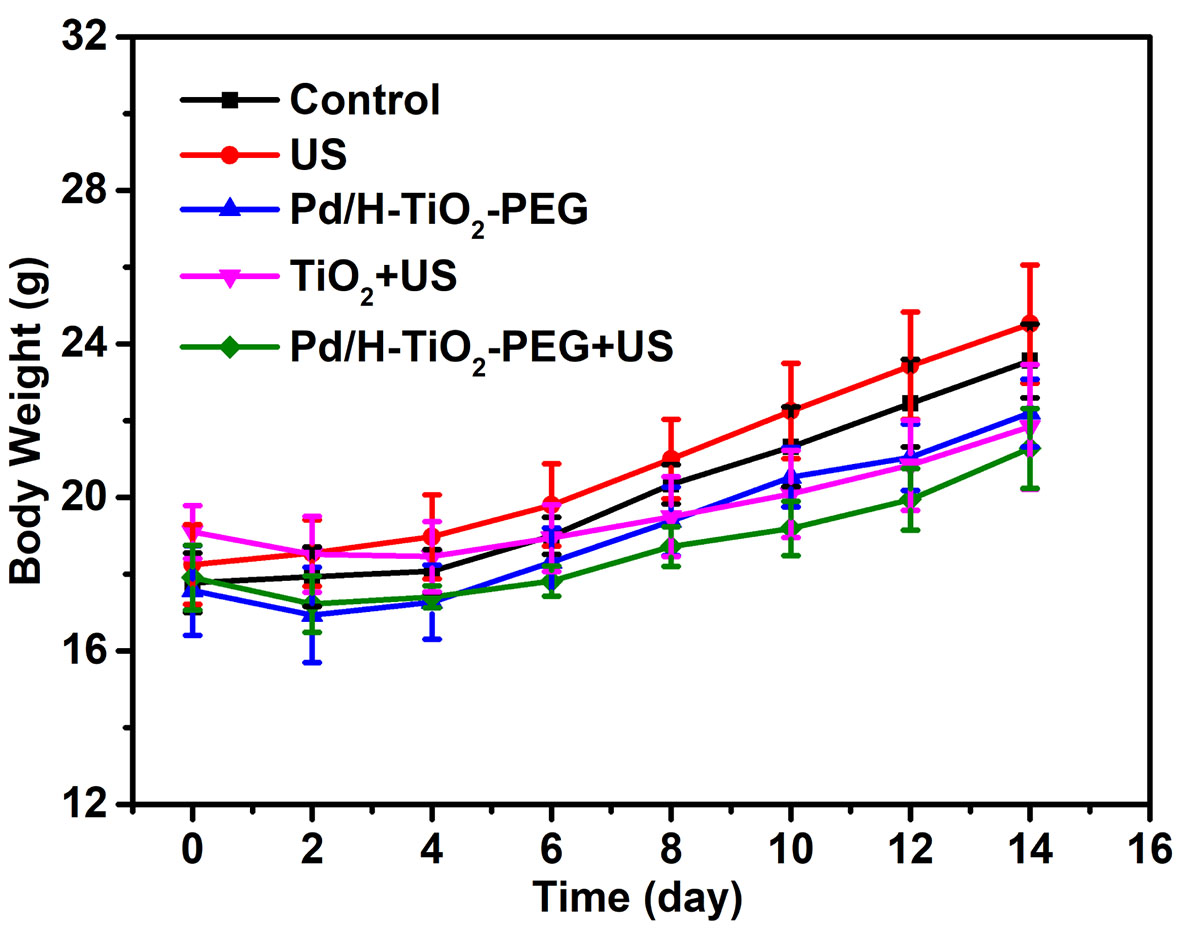


**Fig. S16.** Body weight curves of mice in all groups.


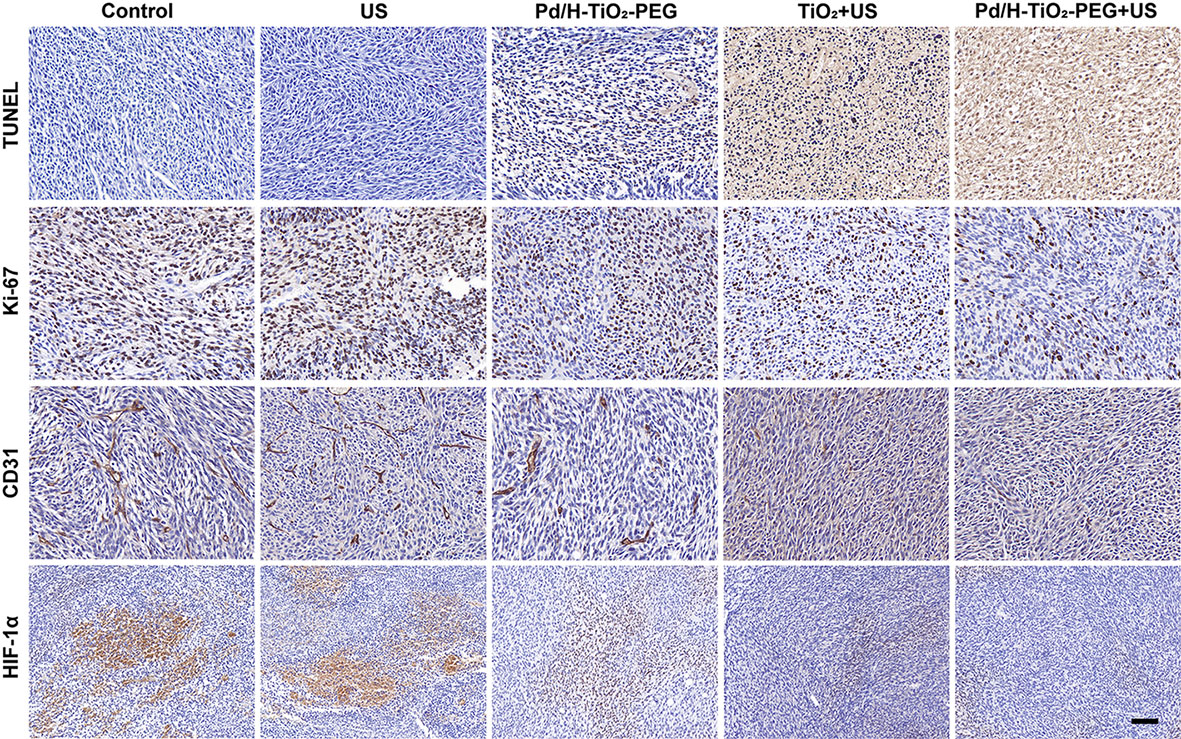


**Fig. S17.** Immunohistochemistry staining images of TUNEL, Ki-67, CD31 and HIF-1α of the tumor tissues after various treatments. Scale bars: 100 μm.


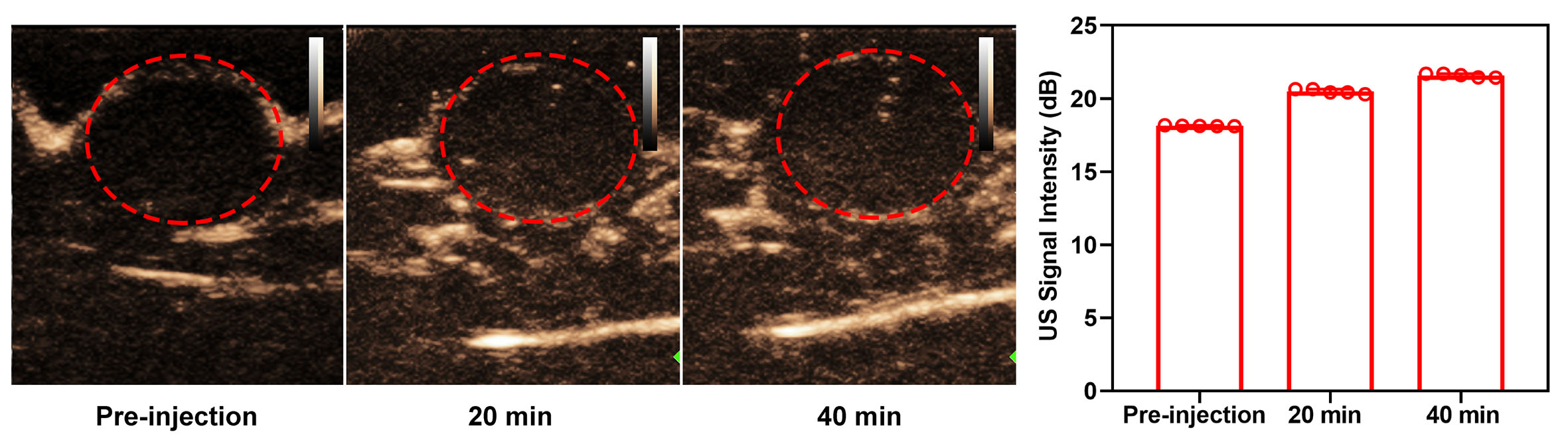


**Fig. S18.** *In vivo* CEUS images of the tumor before and after intratumoral injection of Pd/H-TiO_2_-PEG, and the corresponding signal intensities.


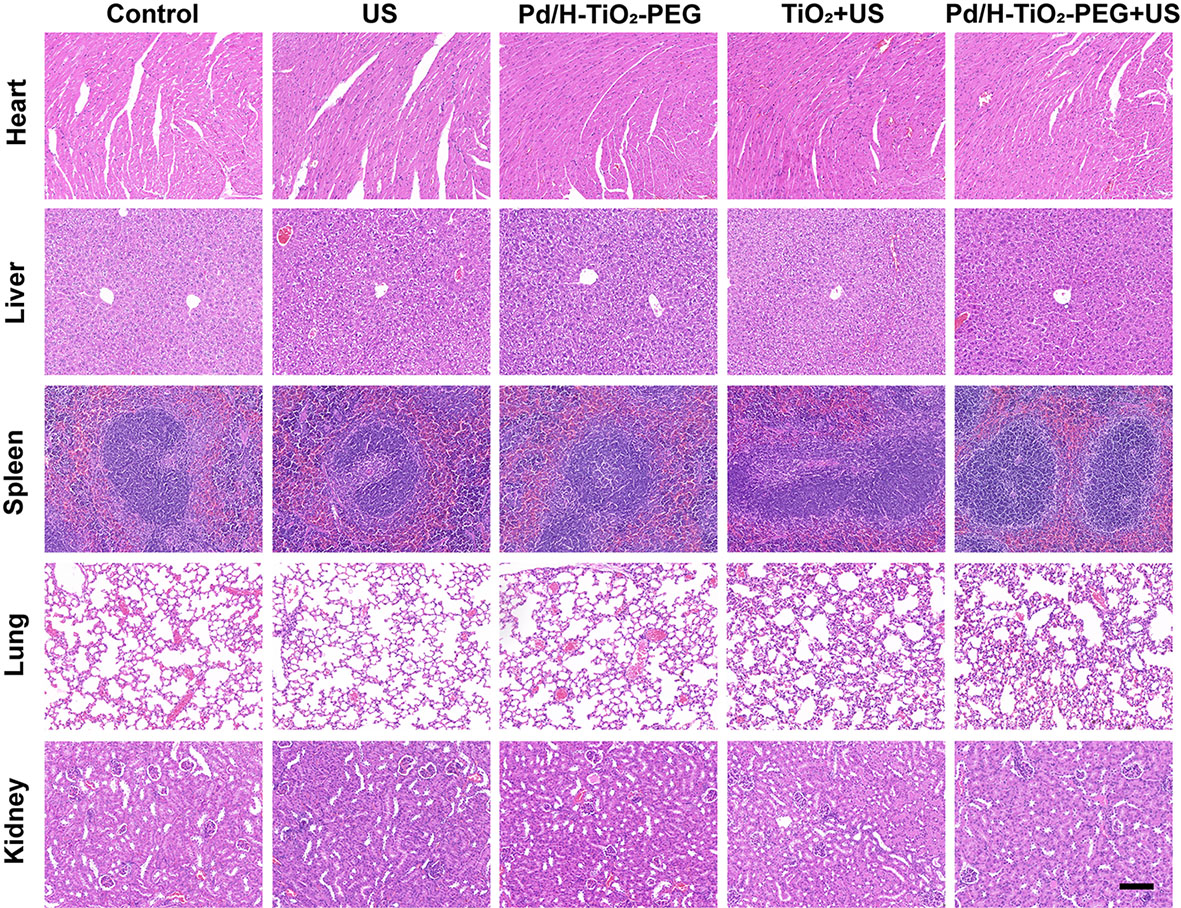


**Fig. S19.** H&E staining images of the major organs of C6 tumor-bearing mice after various treatments. Scale bars: 100 μm.

**References**

1. Samat MH, Ali AMM, Taib MFM, Hassan OH, Yahya MZA. Structural and electronic properties of TiO_2_ polymorphs with effective on-site coulomb repulsion term: DFT+U approaches. Mater Today Proc. 2019; 17: 472-483.
2. Liu R, Yang F, Xie Y, Yu Y. Visible-light responsive boron and nitrogen codoped anatase TiO_2_ with exposed {001} facet: Calculation and experiment. Appl Surf Sci. 2019; 466: 568-577.
3. Nilsing M, Lunell S, Persson P, Ojamäe L. Phosphonic acid adsorption at the TiO_2_ anatase (101) surface investigated by periodic hybrid HF-DFT computations. Surf Sci. 2005; 582: 49-60.
4. Livraghi S, Paganini MC, Giamello E, Selloni A, Valentin CD, Pacchioni G. Origin of photoactivity of nitrogen-doped titanium dioxide under visible light. J Am Chem Soc. 2006; 128: 15666–15671.
5. Muscat J, Wander A, Harrison NM. On the prediction of band gaps from hybrid functional theory. Chem Phys Lett. 2001; 342: 397-401.
6. Kresse G, Hafner J. Ab initio molecular dynamics for open-shell transition metals. Phys Rev B. 1993; 48: 13115-13118.
7. Grimme S. Semiempirical GGA-type density functional constructed with a long-range dispersion correction. J Comput Chem. 2006; 27: 1787-1799.
8. Perdew JP, Burke K, Ernzerhof M. Generalized gradient approximation made simple. Phys Rev Lett. 1996; 77: 3865-3868.
